# Enhanced hippocampal type II theta activity AND altered theta architecture in mice lacking the Ca_v_3.2 T-type voltage-gated calcium channel

**DOI:** 10.1038/s41598-020-79763-4

**Published:** 2021-01-13

**Authors:** Muhammad Imran Arshaad, Magdalena Elisabeth Siwek, Christina Henseler, Johanna Daubner, Dan Ehninger, Jürgen Hescheler, Agapios Sachinidis, Karl Broich, Anna Papazoglou, Marco Weiergräber

**Affiliations:** 1 Experimental Neuropsychopharmacology, Federal Institute for Drugs and Medical Devices (Bundesinstitut für Arzneimittel und Medizinprodukte, BfArM), Kurt-Georg-Kiesinger-Allee 3, 53175 Bonn, Germany; 2 Molecular and Cellular Cognition, German Center for Neurodegenerative Diseases (Deutsches Zentrum für Neurodegenerative Erkrankungen, DZNE), Sigmund-Freud-Str. 27, 53127 Bonn, Germany; 3 Institute of Neurophysiology, University of Cologne, Faculty of Medicine, Robert-Koch-Str. 39, 50931 Cologne, Germany; 4 Federal Institute for Drugs and Medical Devices (Bundesinstitut für Arzneimittel und Medizinprodukte, BfArM), Kurt-Georg-Kiesinger-Allee 3, 53175 Bonn, Germany

**Keywords:** Neuroscience, Physiology

## Abstract

T-type Ca^2+^ channels are assumed to contribute to hippocampal theta oscillations. We used implantable video-EEG radiotelemetry and qPCR to unravel the role of Ca_v_3.2 Ca^2+^ channels in hippocampal theta genesis. Frequency analysis of spontaneous long-term recordings in controls and Ca_v_3.2^−/−^ mice revealed robust increase in relative power in the theta (4–8 Hz) and theta-alpha (4–12 Hz) ranges, which was most prominent during the inactive stages of the dark cycles. Urethane injection experiments also showed enhanced type II theta activity and altered theta architecture following Ca_v_3.2 ablation. Next, gene candidates from hippocampal transcriptome analysis of control and Ca_v_3.2^−/−^ mice were evaluated using qPCR. Dynein light chain Tctex-Type 1 (Dynlt1b) was significantly reduced in Ca_v_3.2^−/−^ mice. Furthermore, a significant reduction of GABA A receptor δ subunits and GABA B1 receptor subunits was observed in the septohippocampal GABAergic system. Our results demonstrate that ablation of Ca_v_3.2 significantly alters type II theta activity and theta architecture. Transcriptional changes in synaptic transporter proteins and GABA receptors might be functionally linked to the electrophysiological phenotype.

## Introduction

Hippocampal theta oscillations of species specific frequency are of major significance in various cognitive and behavioral processes, such as arousal, attention, exploratory behavior, voluntary locomotion, learning and memory, sensorimotor processing and REM sleep^1–6^. Anatomically, the minimal substrate for triggering and perpetuation of theta activity turned out to be the hippocampus and the medial septum-diagonal band of Broca (MS/DBB)^7–9^. The exact locality determination of the theta generator turned out to be challenging and both extrahippocampal and intrahippocampal hypotheses are under discussion^10^. Nowadays, the so-called *septal pacemaker-hippocampal follower model* is widely accepted, based on studies by Hangya et al. who showed that a subgroup of GABAergic medial septum (MS) neurons operates as the pacemaker structure by forwarding rhythmic activity on hippocampal pyramidal cells and interneurons^11^. Based on the dualistic theory of theta oscillations, one can distinguish between atropine-insensitive type I and atropine-sensitive type II theta activity^12–14^. However, the molecular, electrophysiological, and behavioral characteristics of hippocampal theta oscillations are still poorly understood^12,15,16^. Atropine-sensitive type II theta oscillations occur, i.a., during alert immobility and urethane induced anesthesia^12,13,17,18^. They can be triggered by stimulation of muscarinic type 1/3 (M_1_/M_3_) G-protein coupled receptors (GPCR), initializing the muscarinic signal transduction cascade via the G protein q/11 alpha subunit (Gα_q/11_), phospholipase C β_1/4_ (PLCβ_1/4_), inositol trisphosphate (InsP_3_), diacylglycerole (DAG), Ca^2+^ and protein kinase C (PKC)^14,19^. Various downstream effects of this cascade, potentially associated with the development of atropine-sensitive type II theta oscillations, have been suggested^14^. Importantly, inactivation of hippocampal PLCβ_1_ or septal PLCβ_4_ resulted in total loss or major mitigation of synchronized theta oscillations^19,20^. Based on their cellular and subcellular expression pattern and functional contribution to dendritic resonance phenomena, voltage-gated Ca^2+^ channels (VGCCs) represent key components in theta genesis though the exact mechanisms remain to be determined^21,22^. One VGCC entity proven to be involved in theta genesis is the high- to mid voltage-activated (HVA, MVA) Ca_v_2.3 R-type Ca^2+^ channel^14,23–25^. Tai et al. early suggested that M_1_/M_3_ muscarinic acetylcholine receptor (mAChR) activation via carbachol can trigger synchronized hippocampal theta oscillations through the G_q/11_, PLCβ_1_, and PKC-mediated stimulation of Ca_v_2.3 R-type VGCCs^26–29^. Importantly, theta oscillatory activity is also sensitive to divalent heavy metal ions such as nickel (Ni^2+^) which potently blocks Ca_v_2.3 Ca^2+^ channels^26,30^. Notably, low micromolar concentrations of Ni^2+^ also substantially inhibit low voltage-activated (LVA) T-type VGCCs. Thus, it remained unclear how and to which extend LVA T-type Ca^2+^ channels are involved in theta genesis. Three subtypes of T-type channels have been cloned, i.e., Ca_v_3.1 (α_1_G), Ca_v_3.2 (α_1_H) and Ca_v_3.3 (α_1_I)^31,32^. They are widely expressed throughout the brain and serve the internal fine tuning of intracellular Ca^2+^ homeostasis, gene regulation and neuronal excitability^33–37^. Furthermore, T-type Ca^2+^ channels are involved in action potential generation, Ca^2+^-dependent low-threshold currents and related rhythmic burst-firing patterns, neurotransmitter release and synaptic plasticity^31,33,38^. They are also involved in several other physiological processes including sleep architecture, body weight maintenance and regulation of pain^39–42^. Disruption of T-type VGCCs has been associated with a number of neuropsychiatric disorders such as epilepsy, insomnia, depression, schizophrenia, Parkinson’s disease and chronic pain syndromes^38^. Recently, Gangadharan et al. investigated theta activity in global Ca_v_3.1^−/−^ mice and mice with specific knockdown of the Ca_v_3.1 gene in the MS, focusing on potential neural mechanisms underlying exploratory behavior. Selective Ca_v_3.1 inactivation in the MS augmented object exploration, whereas global Ca_v_3.1 inactivation resulted in both enhanced-object and open-field exploration^43^. Notably, only type II hippocampal theta was enhanced in the MS knockdown animals, whereas both type I and type II theta rhythms were increased in global Ca_v_3.1^−/−^ mice. This specific effect is potentially related to a strong increase in excitability of septohippocampal GABAergic neurons and a shift from the burst to the tonic firing pattern^43^. Importantly, other T-type Ca^2+^ channels also exhibit strong expression in the septohippocampal system, particularly Ca_v_3.2 which is co-expressed with Ca_v_3.1 and sometimes even expressed at higher levels in structures related to theta genesis^44^. In general, immunoreactivity for Ca_v_3.2 is more prominent in the brain than for Ca_v_3.1. In the hippocampus, strong reactivity for Ca_v_3.2 was detected in pyramidal neurons and interneurons with a complex spatial distribution pattern on the dendritic/somatic level and the septohippocampal network level in general^44^. The expression pattern of Ca_v_3.2 suggests a complex involvement in theta genesis. Previous studies have shown that Ca_v_3.2 Ca^2+^ channels are crucial for hippocampal long-term potentiation (LTP), cued-context fear conditioning tasks and passive avoidance strategies^45^. Deletion of Ca_v_3.2 was further reported to promote anxiety-related behavior, to impair learning and memory and to cause reduced sensitivity to psychostimulants^46^. The present study was carried out to unravel the role of Ca_v_3.2 T-type Ca^2+^ channels in initiation, maintenance, and modulation of hippocampal theta oscillations and the underlying molecular and electrophysiological mechanisms.

## Results

### Experimental design and representative EEG traces from Ca_v_3.2^+/+^ and Ca_v_3.2^−/−^ mice

**Figure 1:**
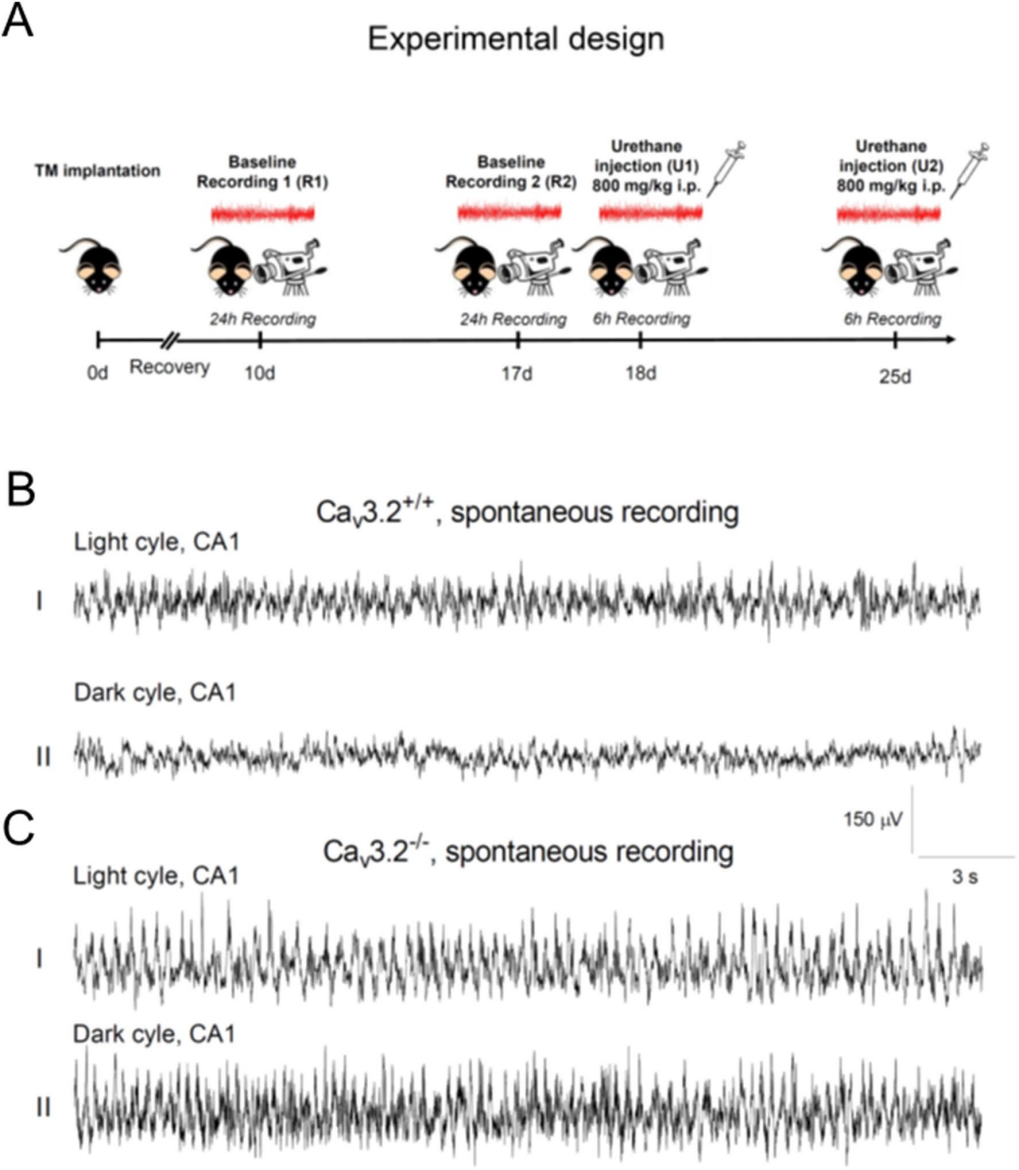
Experimental design and hippocampal EEG recordings from Ca_v_3.2^+/+^ and Ca_v_3.2^−/−^ mice. (**A**) Experimental design including EEG radiofrequency transmitter (TM) implantation (day 0), a 10 days recovery period, two 24 h EEG long-term recordings (R1 at day 10, R2 at day 17) and two 6 h EEG recordings following urethane injection (800 mg/kg i.p., U1 at day 18, U2 at day 25). (**B**,**C**) Representative 30 s EEG traces from the CA1 region for the light (**B**_**I**_,**C**_**I**_) and dark cycle (**B**_**II**_,**C**_**II**_) R1 long-term recording from Ca_v_3.2^+/+^ (**B**) and Ca_v_3.2 deficient mice (**C**). Note that Ca_v_3.2^−/−^ mice display prominent theta/alpha activity compared to Ca_v_3.2^+/+^ animals, particularly during the inactive state (see “Results” section). Scale: y-axis, 150 μV; x-axis, 3 s.

Ca_v_3.2^+/+^ and Ca_v_3.2 deficient mice were implanted with a radiofrequency transmitter at day 0. After a 10 days recovery period, the first 24 h baseline recording (R1) was carried out, followed by a second 24 h long-term recording on day 17 (R2) (Fig. 1A). Both recordings were analyzed for activity, temperature and relative EEG power for the individual frequency bands. Subgroup specific analysis, including circadian rhythmicity (light/dark cycle), activity state and genotype was also performed. Following baseline recordings, type II theta oscillations were induced pharmacologically by injection of urethane (800 mg/kg i.p., U1, U2) (Fig. 1A).

Representative 30 s EEG recordings from the CA1 hippocampal area of both Ca_v_3.2^+/+^ and Ca_v_3.2^−/−^ mice are displayed in Fig. 1B,C. Recordings from Ca_v_3.2^+/+^ mice show, i.a., typical large irregular amplitude (LIA) activity, e.g., during the light cycle (Fig. 1B_I_) whereas there is a general enhancement in EEG frequency during the dark cycle (Fig. 1B_II_). In Ca_v_3.2 deficient mice, theta and alpha activity is enhanced, particularly during the non-active dark cycle (Fig. 1C_II_, see also Figs. 5B,C and 7B,C below).

### Activity profile in Ca_v_3.2^+/+^ and Ca_v_3.2^−/−^ mice

**Figure 2:**
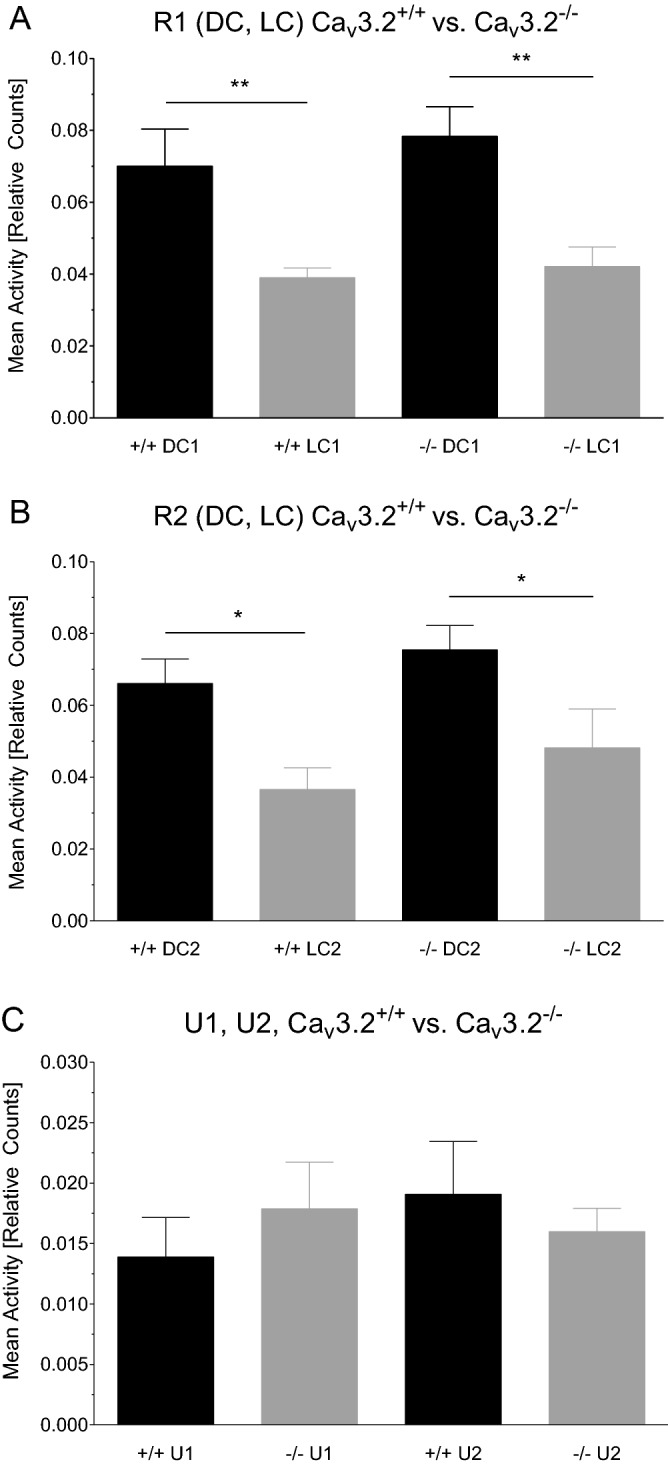
Activity profile in Ca_v_3.2^+/+^ and Ca_v_3.2^−/−^ mice. Activity profile of Ca_v_3.2^+/+^ and Ca_v_3.2^−/−^ mice during the light cycle (LC1) and dark cycle (DC1) for the first (R1, **A**) and second (LC2, DC2, R2, **B**) 24 h long-term recording. Both genotypes exhibit significantly increased motor activity during the DC compared to the LC in both long-term recordings (R1, R2) resembling the nocturnal behavioral characteristics of mice. No differences were observed between both genotypes, neither in R1 nor in R2. (**C**) Activity values following both urethane injections (U1, U2, 800 mg/kg i.p. each) exhibiting no significant differences within and between the genotypes. Compared to (**A**) and (**B**), mean activity values are reduced as the multi-target drug urethane does not only induce hippocampal type II theta activity but also acts as a sedative due to its antagonistic effects at glutamate receptors. Note that mean activity data represent averaged relative counts from 2 s epochs.

The mean activity for the dark cycle (DC, 12 h) and light cycle (LC, 12 h) of the first (R1) and second (R2) 24 h long-term recording was analyzed for both genotypes. Note that activity parameters provided by the telemetry system represent relative counts in the horizontal plane. Mice are nocturnal animals with predominant activity and locomotion in the DC. Consequently, Ca_v_3.2^+/+^ exhibit significantly increased activity during DC1 compared to LC1 (0.070 ± 0.010 (DC1) vs. 0.039 ± 0.003 (LC1), p = 0.0084). The same holds true for Ca_v_3.2^−/−^ mice (0.078 ± 0.008 (DC1) vs. 0.042 ± 0.005 (LC1), p = 0.0034) (Fig. 2A). No significant changes were observed between both genotypes for either DC1 or LC1. Importantly, the same circadian architecture in locomotion was observed for the second 24 h baseline recording (R2). Again, Ca_v_3.2^+/+^ mice displayed increased relative activity in DC2 compared to LC2 (0.066 ± 0.007 (DC2) vs. 0.037 ± 0.006 (LC2), p = 0.0104). The same holds true again for Ca_v_3.2^−/−^ mice (0.075 ± 0.007 (DC2) vs. 0.048 ± 0.011 (LC2), p = 0.0382) (Fig. 2B). In summary, both genotypes showed a comparable circadian activity profile which is important for interpretation of results from relative EEG power analysis outlined below.

The activity profile was also analyzed post urethane injection for 6 h (U1, U2). As becomes apparent in Fig. 2C, no significant alterations between both genotypes could be detected (U1, 0.014 ± 0.003 (Ca_v_3.2^+/+^) vs. 0.018 ± 0.004 (Ca_v_3.2^−/−^), p = 0.4433; U2, 0.019 ± 0.004 (Ca_v_3.2^+/+^) vs. 0.016 ± 0.002 (Ca_v_3.2^−/−^), p = 0.5281). Note that urethane does not only induce hippocampal type II theta oscillations but also hypolocomotion based on its anti-glutamatergic effects^47^.

### Temperature profile in Ca_v_3.2^+/+^ and Ca_v_3.2^−/−^ mice

**Figure 3:**
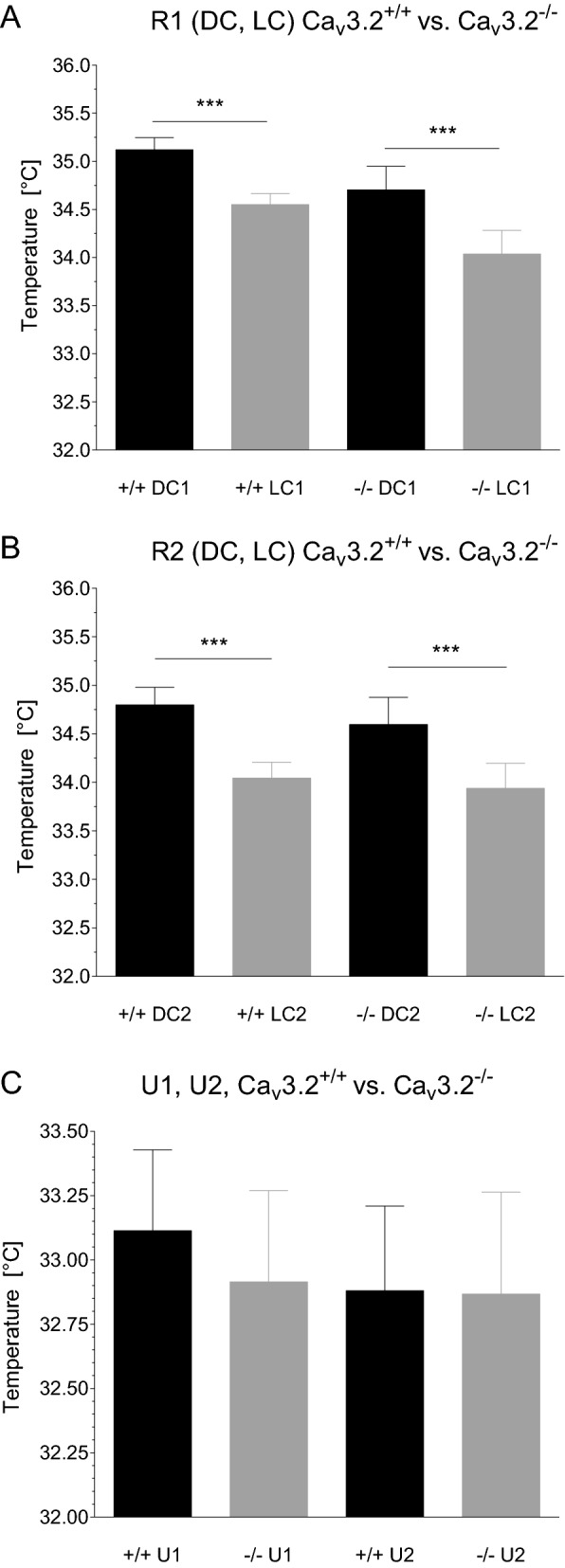
Temperature profile of Ca_v_3.2^+/+^ and Ca_v_3.2^−/−^ mice during the light cycle (LC) and dark cycle (DC) for the first (R1, **A**) and second (R2, **B**) 24 h long-term recording. Significantly increased motor activity during the DC (Fig. 2A,B) correlates with the circadian pattern of temperature profile in both genotypes during R1 and R2 recordings exhibiting significant differences as well. Note that the TA10ETA-F20 radiofrequency transmitters are placed subcutaneously and that averaged subcutaneous temperature values do not reflect body core temperature. However, under controlled environmental conditions, subcutaneous temperature profiles reliably parallel body core values. (**C**) Temperature values following both urethane injections (U1, U2, 800 mg/kg i.p. each) exhibited no significant differences within and between both genotypes. Compared to (**A**) and (**B**), mean temperature values are reduced due to hypolocomotion following urethane injection.

Besides biopotentials and activity, the TA10ETA-F20 transmitter is capable of recording body temperature. The latter were averaged for the DC and LC for both genotypes. As expected, temperature values in Ca_v_3.2^+/+^ and Ca_v_3.2^−/−^ mice mimicked those results obtained for activity (Figs. 2A,B, 3A,B). As mice are nocturnal animals, both genotypes exhibited significant temperature increase during the DC1 compared to the LC1 of baseline recording R1 (Ca_v_3.2^+/+^: 35.12 ± 0.13 °C (DC1) vs. 34.55 ± 0.11 °C (LC1), p < 0.0001; Ca_v_3.2^−/−^: 34.70 ± 0.24 °C (DC1) vs. 34.04 ± 0.25 °C (LC1), p = 0.0006) (Fig. 3A). No significant differences were observed within the DC1 and LC1 between both genotypes. These findings again mirror the results obtained from the activity study (Fig. 2), pointing out that no significant alterations in activity architecture exists between Ca_v_3.2^+/+^ and Ca_v_3.2^−/−^ mice. Temperature analysis of R2 confirmed results from R1 with a significant increase in DC2 compared to LC2 in both genotypes (Ca_v_3.2^+/+^: 34.80 ± 0.18 °C (DC2) vs. 34.04 ± 0.16 °C (LC2), p = 0.0002; Ca_v_3.2^−/−^: 34.60 ± 0.28 °C (DC2) vs. 33.94 ± 0.26 °C (LC2), p < 0.0001) (Fig. 3B).

Similar to the activity results post urethane injections, no alterations were observed in temperature profile in U1 (33.11 ± 0.31 °C (Ca_v_3.2^+/+^) vs. 32.91 ± 0.36 °C (Ca_v_3.2^−/−^), p = 0.6853) and U2 (32.88 ± 0.33 °C (Ca_v_3.2^+/+^) vs. 32.87 ± 0.40 °C (Ca_v_3.2^−/−^), p = 0.9809) (Fig. 3C).

### FFT based frequency analysis from spontaneous EEG long-term recordings in Ca_v_3.2^+/+^ and Ca_v_3.2^−/−^

In both Ca_v_3.2^+/+^ and Ca_v_3.2 deficient mice, two 24 h long-term (baseline) EEG recordings (R1, R2) from the CA1 region were carried out at day 10 and day 17 post implantation of the TA10ETA-F20 transmitter (Fig. 1A). This regime guarantees a recovery period of 10 days, sufficient for the animals to regain standard physiological parameters, i.a., in CNS electrophysiology and circadian patterns^48^. Subsequently, an FFT based EEG frequency analysis was performed distinguishing between the LC and DC as well as the non-active (inactive) state (NAS) and active state (AS). EEG power values are presented as relative values (%).

#### EEG power analysis of R1/R2 during the active state of the light cycle

**Figure 4:**
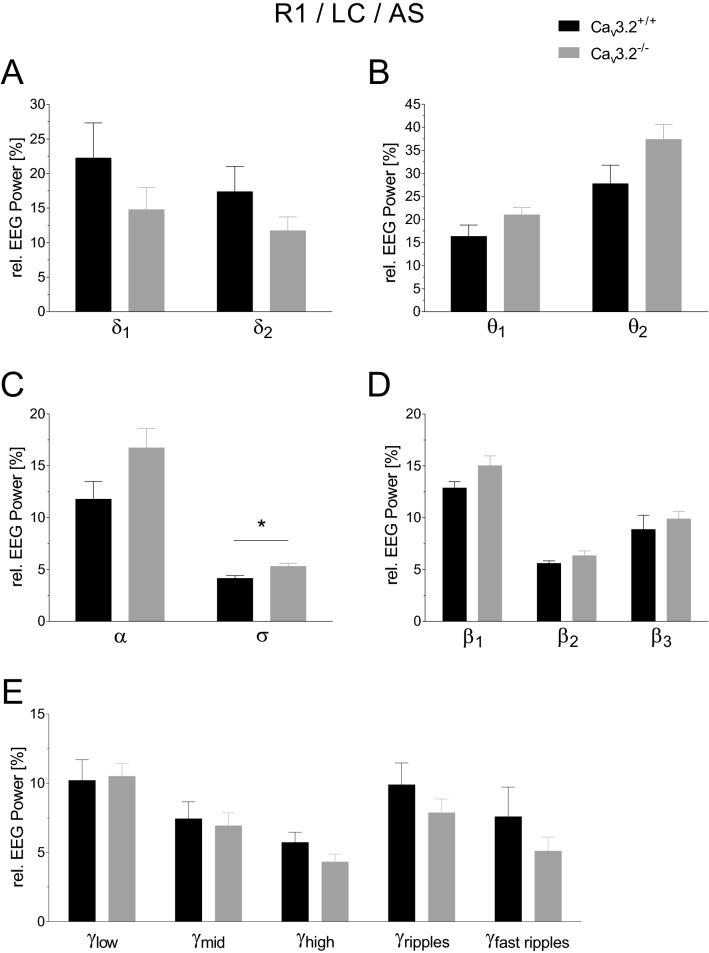
EEG power analysis during the active light cycle state (24 h long-term recording R1) in Ca_v_3.2^+/+^ and Ca_v_3.2^−/−^ mice. Relative EEG power (%) for both genotypes is displayed for the individual frequency ranges (**A–E**). A significant change was observed for σ.

Relative EEG power analysis during the LC, active state (AS) of R1 showed a significant change in σ (4.143 ± 0.279% (Ca_v_3.2^+/+^) vs. 5.300 ± 0.286% (Ca_v_3.2^−/−^), p = 0.0118) (Fig. 4C). No significant alterations were observed for δ_1_, δ_2_, α, θ_1_, θ_2_, β_2_, β_3_ and the γ frequency bands in R1 (Fig. 4A–E).

Analysis of the second baseline recording (R2) however, revealed a significant alteration in relative α and σ power (α, 11.481 ± 1.925% (Ca_v_3.2^+/+^) vs. 18.258 ± 2.341% (Ca_v_3.2^−/−^), p = 0.0421; σ, 4.090 ± 0.472% (Ca_v_3.2^+/+^) vs. 5.398 ± 0.356% (Ca_v_3.2^−/−^), p = 0.0441) (Supplementary Fig. 1B,C). No significant differences were observed for δ_1_, δ_2_, θ_1_, θ_2_, β_1_, β_2_, β_3_ and the various γ frequency bands (Supplementary Fig. 1A,B,D,E).

#### EEG power analysis of R1/R2 during the non-active state of the light cycle

**Figure 5:**
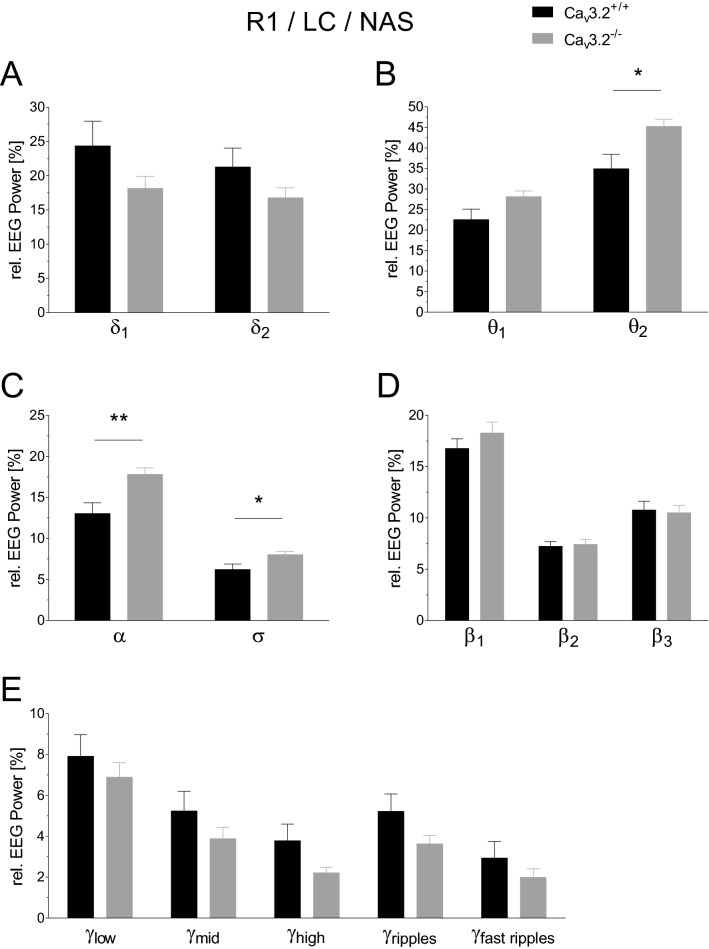
EEG power analysis during the non-active light cycle state (24 h long-term recording R1) in Ca_v_3.2^+/+^ and Ca_v_3.2^−/−^ mice. Relative EEG power (%) for both genotypes is displayed for the individual frequency ranges (**A–E**). Significant alterations were observed for the θ_2_, α and σ frequency ranges (**B**,**C**). Changes in relative theta/theta-alpha power in Ca_v_3.2^−/−^ mice during the NAS point to functional alterations in type II theta activity.

Relative EEG power analysis during the LC, non-active state (NAS) of R1 revealed significant changes in θ_2_, α and σ (θ_2_, 34.928 ± 3.521% (Ca_v_3.2^+/+^) vs. 45.252 ± 1.730% (Ca_v_3.2^−/−^), p = 0.0197; α, 13.033 ± 1.309% (Ca_v_3.2^+/+^) vs. 17.795 ± 0.794% (Ca_v_3.2^−/−^), p = 0.007; σ, 6.208 ± 0.684% (Ca_v_3.2^+/+^) vs. 8.039 ± 0.383% (Ca_v_3.2^−/−^), p = 0.0348) in Ca_v_3.2 deficient mice compared to Ca_v_3.2^+/+^ (Fig. 5B,C). No significant differences could be detected for δ_1_, δ_2_, θ_1_, β_1_, β_2_, β_3_, and the various γ frequency bands between both genotypes for R1 (Fig. 5A,B,D,E). The results obtained from R1 resembled those obtained from R2 EEG recordings. Significant alterations between both genotypes were detected again in R2 for the θ_2_ and α relative power (θ_2_, 34.707 ± 4.035% (Ca_v_3.2^+/+^) vs. 46.247 ± 1.594% (Ca_v_3.2^−/−^), p = 0.0187; α, 12.671 ± 1.622% (Ca_v_3.2^+/+^) vs. 18.189 ± 0.718% (Ca_v_3.2^−/−^), p = 0.0077) (Supplementary Fig. 1B,C). No significant alterations were found for δ_1_, δ_2_, θ_1_, σ, β_1_, β_2_, β_3_, and the various γ frequency bands (Supplementary Fig. 2A–E). Overall, these findings point to an increase in type II theta activity in Ca_v_3.2^−/−^ mice, as the latter is most prominent during alert immobility in the non-active state.

#### EEG power analysis of R1/R2 during the active state of the dark cycle

**Figure 6:**
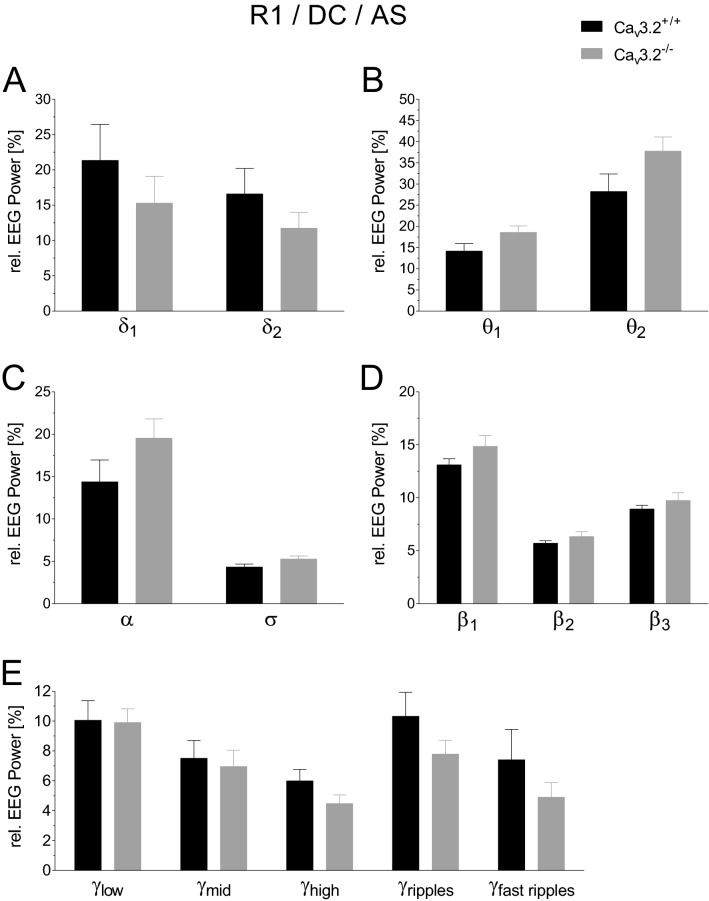
EEG power analysis during the active dark cycle state (24 h long-term recording R1) in Ca_v_3.2^+/+^ and Ca_v_3.2^−/−^ mice. Relative EEG power (%) for the Ca_v_3.2^+/+^ and Ca_v_3.2^−/−^ is displayed for the individual frequency ranges (**A**–**E**). No significant alterations were detected.

Relative EEG power analysis in Ca_v_3.2^+/+^ and Ca_v_3.2^−/−^ mice revealed no significant differences in δ_1_, δ_2_, θ_1_, θ_2_, α, σ, β_1_, β_2_, β_3_, and the γ frequency bands during R1 at the DC in the AS (Fig. 6). As for baseline recording R1, no alterations were observed for the other frequency bands (Supplementary Fig. 3).

#### EEG power analysis of R1/R2 during the non-active state of the dark cycle

**Figure 7:**
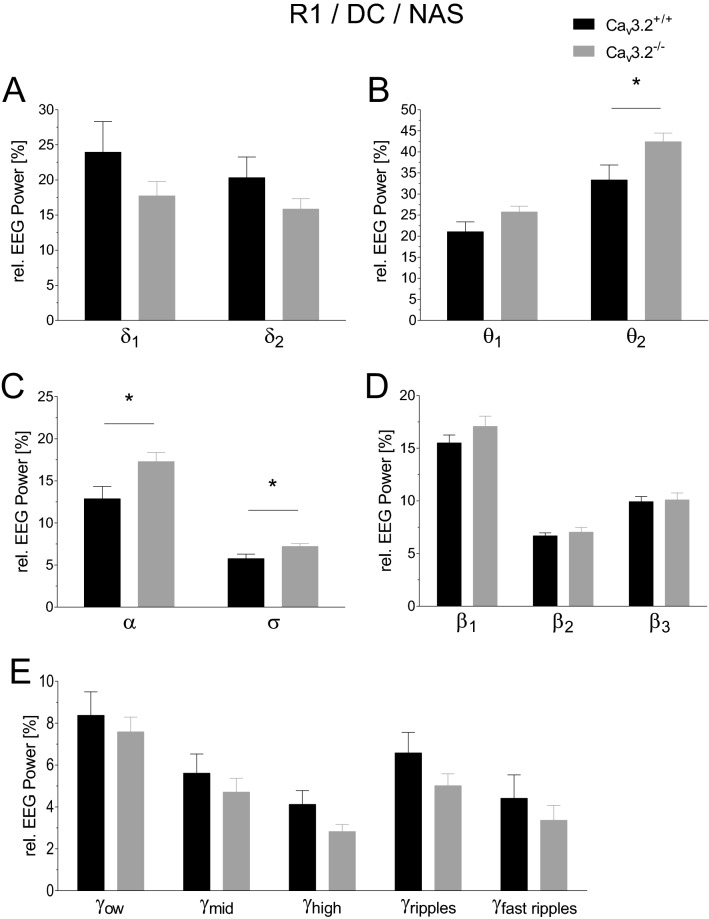
EEG power analysis during the non-active dark cycle state (24 h long-term recording R1) in Ca_v_3.2^+/+^ and Ca_v_3.2^−/−^ mice. Relative EEG power (%) for Ca_v_3.2^+/+^ and Ca_v_3.2^−/−^ animals is displayed for the individual frequency ranges (**A**–**E**). In Ca_v_3.2^−/−^ mice, significant increases were observed in θ_2_, α and σ relative power (**B**,**C**). These alterations point to a functional involvement of Ca_v_3.2 in type II theta activity.

Relative EEG power analysis of the dark cycle, non-active state of R1 exhibited a significant increase in θ_2_, α and σ in Ca_v_3.2^−/−^ mice compared to controls (θ_2_, 33.344 ± 3.553% (Ca_v_3.2^+/+^) vs. 42.415 ± 2.047% (Ca_v_3.2^−/−^), p = 0.0441; α, 12.878 ± 1.458% (Ca_v_3.2^+/+^) vs. 17.287 ± 1.063% (Ca_v_3.2^−/−^), p = 0.0284; σ, 5.762 ± 0.543% (Ca_v_3.2^+/+^) vs. 7.214 ± 0.338% (Ca_v_3.2^−/−^), p = 0.0395) (Fig. 7B,C). No significant alterations were observed for δ_1_, δ_2_, θ_1_, β_1_, β_2_, β_3_, and the γ frequency bands (Fig. 7A,B,D,E). Similar results were detected in the second baseline recording R2. A statistical increase in relative EEG power was again detected in θ_2_ (33.516 ± 4.177% (Ca_v_3.2^+/+^) vs. 44.078 ± 1.882% (Ca_v_3.2^−/−^), p = 0.0370) and α (12.586 ± 1.675% (Ca_v_3.2^+/+^) vs. 18.159 ± 1.030% (Ca_v_3.2^−/−^), p = 0.0132). No significant changes occurred in δ_1_, δ_2_, θ_1_, σ_1_, β_1_, β_2_, β_3_ and the γ frequency bands (Supplementary Fig. 4A–E). These findings again point to an increase in type II theta activity in Ca_v_3.2^−/−^ mice, likely to be related to alert immobility in the non-active state.

In summary, a consistent finding from EEG power analysis turned out to be an increase in θ_2_ and α activity during the non-active state of both the light and dark cycles of R1 and R2 in the in Ca_v_3.2 deficient animals. This alteration in the hippocampal CA1 theta/alpha band resembles those findings observed for Ca_v_3.1 knock-out mice^43^.

### EEG power analysis in Ca_v_3.2^+/+^ and Ca_v_3.2^−/−^ mice following urethane administration

**Figure 8:**
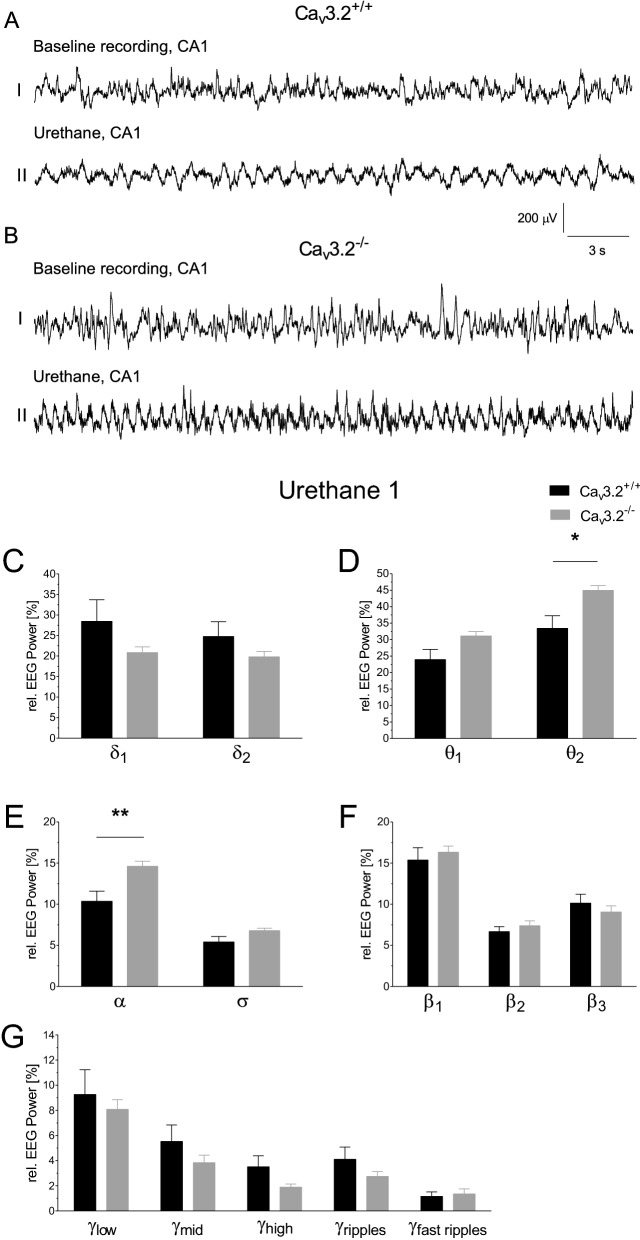
Frequency analysis in Ca_v_3.2^+/+^ and Ca_v_3.2^−/−^ mice following urethane (800 mg/kg i.p.) injection (U1). (**A**) CA1 EEG traces (30 s) from Ca_v_3.2^+/+^ and Ca_v_3.2^−/−^ mice. Prior to urethane injection, baseline recordings (**A**_**I**_,**B**_**I**_) display characteristic large irregular activity (LIA). Following urethane administration (**A**_**II**_,**B**_**II**_), the EEG exhibits prominent theta oscillations in Ca_v_3.2^−/−^ mice (**B**_**II**_). Scale: y-axis, 200 μV; x-axis, 3 s. (**C–G**) Relative EEG power (%) for Ca_v_3.2^+/+^ and Ca_v_3.2^−/−^ mice is displayed for the individual frequency ranges (**A–E**). Urethane which is used to induce atropine sensitive type II theta oscillations, caused a significant increase in θ_2_ and α relative EEG power (**D**,**E**).

Urethane is a multi-target drug that exerts sedative/hypnotic effects leading to hypolocomotion. At higher dosages (~ 2 g/kg i.p.), urethane is used to induce slow-wave sleep. In our study, lower dosages of urethane were used (800 mg/kg i.p.) to induce hippocampal type II theta oscillations^47^. Baseline recordings from the CA1 region from Ca_v_3.2^+/+^ mice (Fig. 8A_I_) and Ca_v_3.2^−/−^ animals (Fig. 8B_I_) display characteristic LIA activity. Urethane is capable of inducing type II theta activity in Ca_v_3.2^+/+^ mice (Fig. 8A_II_) and even more prominent in Ca_v_3.2 deficient animals (Fig. 8B_II_).

Similar to our studies of spontaneous long-term EEG recordings, we performed relative power analysis of post urethane EEGs (U1, U2) in both genotypes at day 18 and day 25 post transmitter implantation. The first urethane administration resulted in significant relative power increase in θ_2_ and α (θ_2_, 33.424 ± 3.807% (Ca_v_3.2^+/+^) vs. 44.941 ± 1.447% (Ca_v_3.2^−/−^), p = 0.0134; α, 10.353 ± 1.232% (Ca_v_3.2^+/+^) vs. 14.615 ± 0.623% (Ca_v_3.2^−/−^), p = 0.0080) (Fig. 8D,E). No significant alterations were observed for δ_1_, δ_2_, θ_1_, σ, and the various β and γ frequencies (Fig. 8C–G). Similar to the results obtained from spontaneous EEG recordings, predominately in the dark, non-active phase, Ca_v_3.2^−/−^ mice exhibited an increase in the theta/alpha range.

To confirm these results, a second urethane injection was carried out at day 25 post transmitter implantation. Again, significant increases in relative power were monitored for θ_1_, θ_2_ and α frequency bands (θ_1_, 24.426 ± 3.129% (Ca_v_3.2^+/+^) vs. 31.973 ± 1.308% (Ca_v_3.2^−/−^), p = 0.0429; θ_2_, 33.650 ± 3.574% (Ca_v_3.2^+/+^) vs. 46.371 ± 1.274% (Ca_v_3.2^−/−^), p = 0.0047; α, 10.204 ± 1.020% (Ca_v_3.2^+/+^) vs. 15.180 ± 0.597% (Ca_v_3.2^−/−^), p = 0.0008) (Supplementary Fig. 5B,C).

As for both 24 h long-term EEG recordings, the urethane studies (U1, U2) clearly confirm an increase in θ_2_ and α activity in the CA1 hippocampal area in Ca_v_3.2 deficient mice.

**Figure 9:**
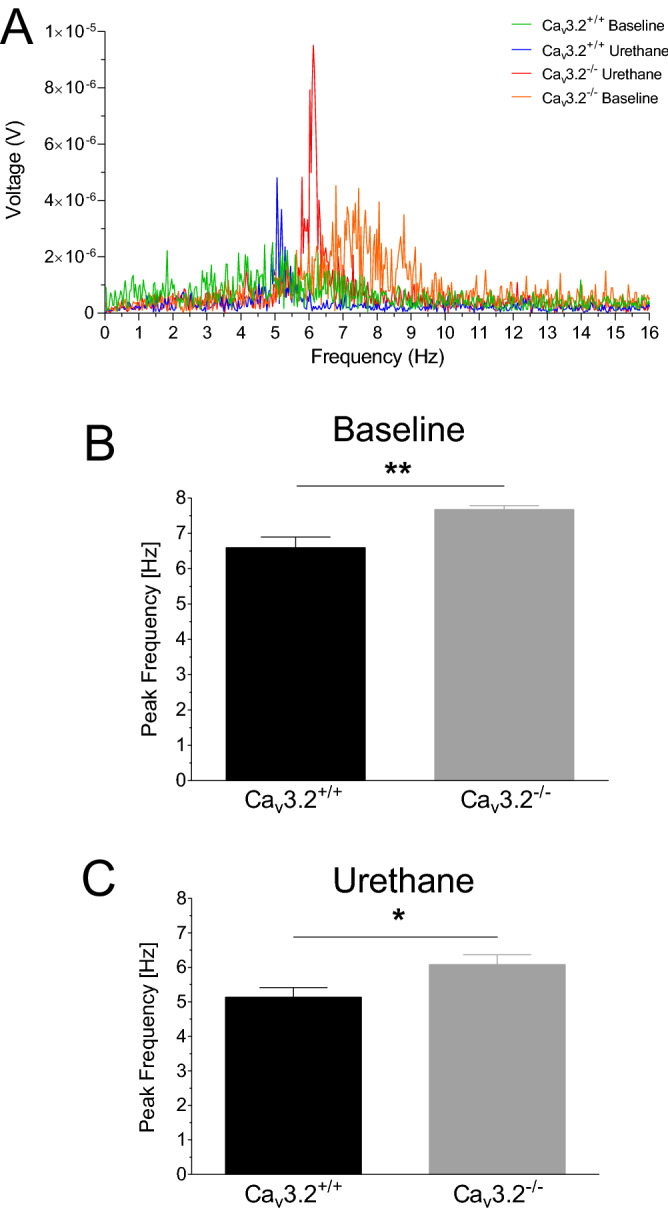
Altered theta architecture in Ca_v_3.2^−/−^ mice. (**A**) Power spectrum density (PSD) plots obtained from representative 30 s EEG traces from baseline and post urethane states from both genotypes. (**B**,**C**) PSD plots from the baseline (**B**) and post urethane state (**C**) were analyzed for peak frequencies in the range of 0–16 Hz. Under both baseline and post urethane conditions, Ca_v_3.2^−/−^ mice exhibited a significant increase in theta peak frequencies.

In order to get a closer insight into the hippocampal theta/alpha architecture of Ca_v_3.2^+/+^ and Ca_v_3.2^−/−^ mice, we analyzed power spectrum density (PSD) plots for theta/alpha peak frequencies. Representative PSD plots for both genotypes from the baseline and post-urethane state are depicted in Fig. 9A. Notably, the peak frequency was increased in Ca_v_3.2^−/−^ mice under baseline conditions (6.598 ± 0.300 Hz (Ca_v_3.2^+/+^) vs. 7.676 ± 0.108 Hz (Ca_v_3.2^−/−^), p = 0.0045, Fig. 9B). The same held true for the post urethane peak frequency (5.134 ± 0.279 Hz (Ca_v_3.2^+/+^) vs. 6.081 ± 0.279 Hz (Ca_v_3.2^−/−^), p = 0.0324, Fig. 9C).

These findings indicate that there is not only an increase in theta/alpha activity in Ca_v_3.2^−/−^ mice but also a shift in theta peak frequency and thus in global theta architecture.

### Transcriptional alterations in the hippocampus of Ca_v_3.2 deficient mice

**Figure 10:**
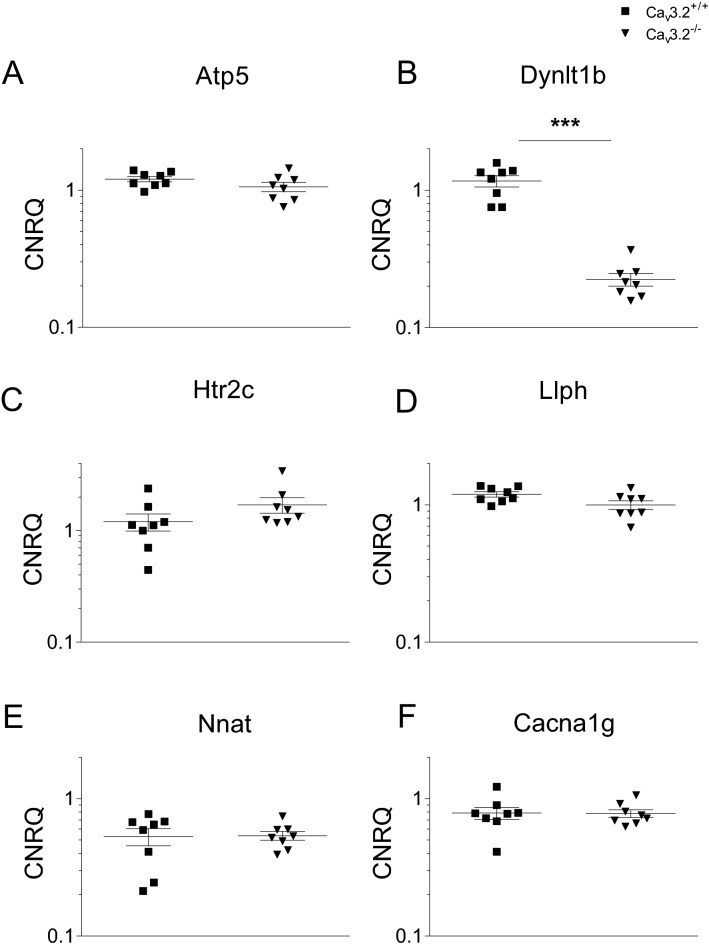

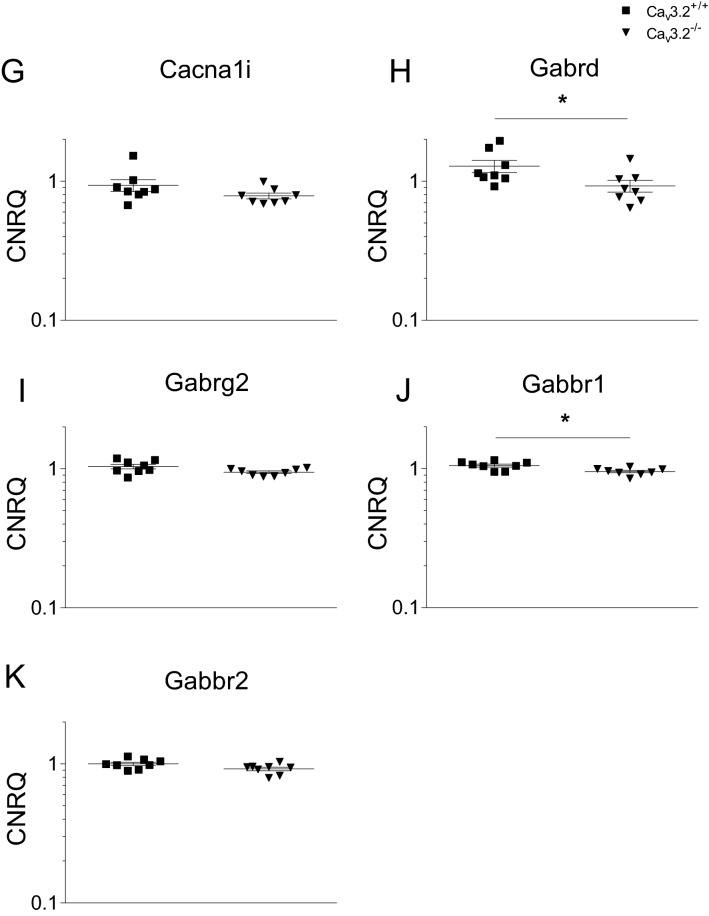
qPCR analysis of candidate genes obtained from hippocampal transcriptome data in Ca_v_3.2^+/+^ and Ca_v_3.2^−/−^ mice. Hippocampal transcriptome experiments were carried out previously. The following gene candidates potentially relevant for hippocampal theta oscillations were analyzed using qPCR: (**A**) ATP synthase, H^+^ transporting, mitochondrial F0 complex, subunit G (Atp5), (**B**) dynein light chain Tctex-Type 1 (Dynlt1b), (**C**) 5-hydroxytryptamine receptor 2C (Htr2c), (**D**) LLP homolog, long-term synaptic facilitation (Aplysia) (Llph), (**E**) Neuronatin (Nnat), (**F**) Ca_v_3.1 (Cacna1g), (**G**) Ca_v_3.3 (Cacna1i), (**H**) GABA A receptor delta subunit (Gabrd), (**I**) GABA A receptor gamma subunit (Gabrg2), (**J**) GABA B1 receptor subunit (Gabbr1), (**K**) GABA B2 receptor subunit (Gabbr2). A significant decrease in transcript levels was observed for Dynlt1b, Gabrd and Gabbr1 in Ca_v_3.2^−/−^ mice (**B**,**H**,**J**).

To gain insight into the mechanisms of theta/alpha augmentation in Ca_v_3.2^−/−^ mice, we performed qPCR analysis of gene candidates (Table 1). These genes were previously detected in a transcriptome analysis from the hippocampi of Ca_v_3.2^+/+^ and Ca_v_3.2^−/−^ mice^49^. Importantly, Ca_v_3.2^−/−^ mice exhibited a significant decrease in transcript levels for dynein light chain Tctex-Type 1 (Dynlt1b) by a fold change (FC) of − 5.208 (p = 0.0002) (Fig. 10B, Table 2).

**Table 1:** Sequences of primer pairs used for qPCR.

Gene	Forward primer (5′–3′)	Reverse primer (5′–3′)
Htr2c^a^	CGGTTCAATTCGCGGACTAAGG	GGTCATTGAGCACGCAGGTAGT
Dynlt1b^a^	GAAGAACGGTGCTGGGTTACAC	CAGATGGACAGTCCGAAGGTAC
Atp5^a^	CCTACAGCTATTCAGAGTGTGAAA	AAAACCACATCCACACCTCAGTG
Nnat^a^	GTGGTGGAGGAAGAGGGTTAAG	CACATTTTGGGGAGGGCTTTCG
Gabrd^b^	TCAAATCGGCTGGCCAGTTCCC	GCACGGCTGCCTGGCTAATCC
Gabrg2^b^	ACTTCTGGTGACTATGTGGTGAT	GGCAGGAACAGCATCCTTATTG
Gabbr1^a^	CGTGGGACTTTTCTATGAGACCG	GAACCAGTTGTCAGCATACCACC
Gabbr2	GGAACACTGCGAAAACACCC	ACCGAACAACATGAGGAGCC
Cacna1g^a^	GACCATGTGGTCCTCGTCATCA	TTTCAGCCAGGAAGACTGCCGT
Cacna1i^a^	GTCTTCACCAAGATGGACGACC	ACTTCGCACCAGTCAGGCTTGT
Llph	TGTTGTCTCTCAGGTGAAGCAT	CCCCGTCCACTCTGAGGATA
Hprt^c^	GCTGGTGAAAAGGACCTCT	CACAGGACTAGAACACCTGC
Actb^b^	GTCCACACCCGCCACCAGTTCG	ATGCCGGAGCCGTTGTCGAC

^a^OriGene Technologies; ^b^Mendu S.K. et al., PLoS One. 2012;7(8):e42959; ^c^Weiergräber M. et al., Basic Res Cardiol. 2005 Jan;100(1):1–13.

**Table 2:** Fold changes of selected microarray gene candidates from Cav3.2^+/+^ and Cav3.2^−/−^ hippocampi.

Gene	Ca_v_3.2^−/−^/Ca_v_3.2^+/+^	
	Fold change	p-value
*Atp5*	− 1.155	0.161
*Dynlt1b*	− 5.208	0.00016
*Htr2c*	1.477	0.065
*Nnat*	1.094	0.798
*Gabrd*	− 1.385	0.015
*Gabrg2*	− 1.092	0.130
*Gabbr1*	− 1.105	0.010
*Gabbr2*	− 1.088	0.083
*Cacna1g*	1.018	0.798
*Cacna1i*	− 1.168	0.130
*Llph*	− 1.211	0.105

CNRQ values are normalized to a calibrator (see “Materials and methods” section) and statistically evaluated using the Mann–Whitney test in qBase plus software.

As the dynein related transportome complex is involved in the transport of GABA receptors, we expanded our qPCR study including the GABA A receptor delta subunit (Gabrd), GABA A receptor gamma subunit (Gabrg2), GABA B1 receptor subunit (Gabbr1) and the GABA B2 receptor subunit (Gabbr2). In Ca_v_3.2^−/−^ mice, a significant decrease was observed for Gabrd (FC, − 1.385; p = 0.015; Fig. 10H, Table 2) and Gabbr1 (FC, − 1.105, p = 0.010, Fig. 10J, Table 2). These findings correlate with the decrease in dynein light chain Tctex-Type 1 transcripts suggesting an overall reduction in the GABA receptor transportome complex and synaptic/extrasynaptic GABA receptor density in the hippocampus, particularly in the hippocampal interneurons.

To exclude that other T-type VGCCs contribute to the theta/alpha phenotype in Ca_v_3.2 deficient mice, we also checked for compensatory alterations in Ca_v_3.1 and Ca_v_3.3 Ca^2+^ channel transcripts. Notably, no changes were observed between both genotypes, which further stresses the idea that the theta/alpha alterations in transgenic mice are due to Ca_v_3.2 ablation itself (Fig. 10, Table 2).

## Discussion

VGCCs play a key role in the generation of theta oscillations in dendrites of hippocampal pyramidal cells^12^, associated with various movement related behaviors^50^, learning tasks and memory processing^51,52^. The septohippocampal circuitry involved in theta genesis acquires innervation from various brain regions to code motor and sensory information processing^6,50,53^, and can trigger the regulation of theta/alpha waves in relation to specific behavioral conditions. Recently, Gangadharan et al. reported that ablation of Ca_v_3.1 VGCCs results in increased theta activity probably based on tonic inhibition of hippocampal GABAergic interneurons via septal GABAergic interneurons. The latter was hypothesized to disinhibit hippocampal pyramidal neurons and to cause increased theta activity^43^.

Although Ca_v_3.1 is prominently expressed in the septohippocampal system, the expression of Ca_v_3.2 clearly predominates^34,44^. Therefore, we investigated the role of Ca_v_3.2 in the generation and architecture of theta/alpha activity in Ca_v_3.2 deficient mice. Previous studies had suggested a complex phenotype upon Ca_v_3.2 ablation including, i.a., impaired memory formation and elevated anxiety^46^. Decreased memory function in Ca_v_3.2^−/−^ mice was originally described using two hippocampal recognition settings, i.e., the novel object recognition (NOR) and spatial object recognition (SOR) testing. The Ca_v_3.2^−/−^ mice did not exhibit preference for the novel or the relocated object compared to wild type animals. Importantly, this altered response was not due to an impairment of the exploratory drives^46^. Interestingly, the spatial working memory remained unaltered in Ca_v_3.2 deficient mice. The same held true for motor skill learning. Furthermore, behavioral studies in the elevated plus maze and open field test strongly underlined a functional implication of Ca_v_3.2 VGCCs in anxiety-related behaviors^46^.

Given these findings and the fact that Ca_v_3.2 VGCC expression outnumbers Ca_v_3.1 expression in the septohippocampal system^44^, we analyzed the role of Ca_v_3.2 in theta genesis and theta architecture relevant for memory formation. Using implantable EEG radiotelemetry from the hippocampal CA1 region and frequency analysis, we elaborated that Ca_v_3.2 Ca^2+^ channels substantially contribute to atropine-sensitive type II theta oscillations and modulate theta architecture. Thus, this is the first direct functional link between Ca_v_3.2 VGCCs and rodent theta oscillation in vivo. Importantly, the significant increase in θ_2_ and α relative EEG power was observed during the NAS of the LC as well as the DC of R1 and R2 and also U1 and U2. The NAS is characterized, i.a., by alert immobility, a physiological state known to exhibit hippocampal type II theta activity. Consequently, theta alterations in Ca_v_3.2^−/−^ mice are likely to be related to atropine-sensitive type II theta. These findings are further confirmed by our urethane injections studies. Pharmacodynamically, urethane serves as a multi-target drug with both agonistic and antagonistic effects on various ligand- and voltage-gated ion channels. Whereas muscarinic and nicotinic AChRs, GABA A receptors, and glycine receptors are stimulated upon urethane injection, NMDA and AMPA receptors are inhibited^54,55^. Urethane is known to induce type II theta activity and Ca_v_3.2^−/−^ mice again revealed an increase in the relative EEG power in the theta/alpha band and the theta peak frequencies in this pharmacological setting. Notably, motor activity can have an important impact on theta I/theta II distribution. It is thus important to stress that both Ca_v_3.2^+/+^ and Ca_v_3.2^−/−^ mice display characteristic circadian activity profiles. No differences in activity were observed between both genotypes indicating that alterations in the hippocampal θ_2_ and α band are not related to alterations in locomotion. It should be noted that besides the consistent changes in the θ_2_ and α frequency bands in R1, R2, U1 and U2, inconsistent alterations were observed for α and σ bands during the LC of the AS in R1 and R2 and for θ_1_ in U1 and U2. No changes were detected for the AS of the DC in R1 and R2.

Anxiety related behavior is another aspect that might influence hippocampal type II theta activity associated with alert immobility. Anxiety analysis in Ca_v_3.2^−/−^ mice using the light/dark conflict test/context^56^ and spontaneous exploratory behavior analysis via open field test and the elevated-plus maze (EPM) suggested increased anxiety in Ca_v_3.2^−/−^ mice not associated with repetitive and compulsive behaviors^46^. Importantly, these results contrast with a previous study from Choi et al. pointing out a lack of anxiety-related behavior in Ca_v_3.2^−/−^ mice using the light/dark conflict test^57^. This apparent discrepancy might be due to the genetic background of the Ca_v_3.2^−/−^ mice^58^ and the behavioral procedure used in the two studies^43,56,57,59–61^. Also, increased anxiety does not necessarily coincide with an increase in theta activity. Ablation of the septal PLCβ_4_ isoform for example caused attenuated type II theta rhythm but increased anxiety^14,19,62^. Thus, θ_2_ and α alterations in Ca_v_3.2 deficient mice do not seem to be attributable to potential changes in anxiety levels.

Next, we investigated the molecular mechanisms underlying the theta/alpha related changes in Ca_v_3.2^−/−^ mice. In general, VGCCs are crucial for LTP, learning and memory functions^63–66^. Disruption of T-type Ca^2+^ channel activity was shown to severely change the induction and maintenance of LTP in the hippocampus, visual cortex and cerebellum^45,67,68^. Furthermore, T-type Ca^2+^ channels interfere with the neurotransmitter release machinery and modulate synaptic transmission^69–71^. Recently, Gangadharan et al. found that Ca_v_3.1^−/−^ mice exhibit increased type II theta activity. This increase was related to a shift in the firing pattern of septal GABAergic interneurons from the burst mode to the tonic mode. LVA T-type Ca^2+^ channels are known to mediate low-threshold Ca^2+^ spikes and burst activity^72–74^. Thus, ablation of Ca_v_3.1 resulted in tonic inhibition of hippocampal GABAergic interneurons via projecting septal GABAergic interneurons. Subsequent perisomatic disinhibition of hippocampal pyramidal neurons was supposed to enhance theta activity in Ca_v_3.1^−/−^ mice^43,75,76^. Given the fact, that Ca_v_3.2 expression outnumbers the expression of Ca_v_3.1 in the septohippocampal system^44^, we hypothesized that Ca_v_3.2 ablation causes a similar sequence of septal GABAergic tonic inhibition and disinhibition of pyramidal cells as observed in Ca_v_3.1^−/−^ mice. To confirm this mechanism of action in Ca_v_3.2^−/−^ mice, we had a closer look at the functional aspects of the septohippocampal system once again. We first investigated the outcomes of our previous transcriptome analysis from the hippocampus of Ca_v_3.2^+/+^ and Ca_v_3.2^−/−^ mice^49^. qPCR analysis revealed a significant reduction in dynein light chain Tctex-Type 1 (Dynlt1b) in Ca_v_3.2^−/−^ mice. The dynein light chain is part of a GABA receptor transportome complex mediating the translocation of GABA receptors to the subsynaptic or extrasynaptic membrane^77–79^. This was a first indication that the GABAergic system is indeed altered in the septohippocampal system of Ca_v_3.2 deficient mice. Therefore, we next analyzed transcript levels of GABA A and GABA B receptors. In Ca_v_3.2^−/−^ mice, transcript levels for the GABA A receptor δ subunit and the GABA B_1_ receptor subunit were significantly reduced. These findings strongly support our GABA hypothesis of enhanced θ_2_/α activity in Ca_v_3.2^−/−^ mice, as GABA A receptor-mediated inhibition within the CNS occurs by fast synaptic transmission and sustained tonic inhibition^80,81^. As observed in dentate gyrus granule cells and thalamic neurons, extrasynaptically located GABA A receptors that contain, e.g., δ subunits, mediate tonic current that is relevant for neuronal/interneuronal excitability in response to ambient GABA concentrations^82–84^. On the other hand, GABA B1-subunit containing receptors can be detected within dendritic spines and mediate slow postsynaptic inhibition^85,86^.

Importantly, we have no indication from our own microarray analysis or qPCR studies that there are compensatory transcriptional alterations of the other T-type Ca^2+^ channels, i.e., Ca_v_3.1 and Ca_v_3.3, in the hippocampus of Ca_v_3.2^−/−^ mice. Thus, the changes observed seem to be solely attributable to Ca_v_3.2 ablation itself.

In summary, our qPCR findings might support the hypothesis that both postsynaptic and extrasynaptic GABA receptors are decreased upon tonic inhibition of hippocampal interneurons and that diminished plasma membrane density is due to an impaired dynein/GABA receptor containing transportome complex. Additional synaptic transporter studies and patch-clamp recordings in hippocampal slices are necessary to directly prove a potential septohippocampal disinhibition in Ca_v_3.2^−/−^ mice. It should also be noted that hippocampal frequency characteristics can be age and gender specific^87–89^. We decided to use males in our study to avoid any potential interference with the estrous cycle in females. Hierarchical fights as a potential source of variability in males^89^, are not relevant in our experimental settings as transmitter implanted mice are housed individually post implantation.

Our study is the first one to prove that Ca_v_3.2 ablation results in increased atropine sensitive type II theta activity and altered theta architecture in the CA1 region. We hypothesize that tonic inhibition of hippocampal GABAergic interneurons and subsequent disinhibition of pyramidal cells due to Ca_v_3.2 ablation might result in compensatory changes in the GABAergic system. These imply both the downregulation of the dynein containing GABA receptor transporter/trafficking complex and GABA A and B receptor complexes themselves. Notably, compensatory changes in other neuronal cell types and circuitries affecting the septohippocampal network cannot be excluded in the gobal Ca_v_3.2 knockout used in our study. Recently, Dinamarca et al.^90^ have shown that GABA B receptors (GBR) form complexes with amyloid precursor protein (APP). This GBR/APP complex is supposed to stabilize APP at the surface membrane and to reduce proteolysis from APP to Aβ. Impaired GABA receptor trafficking and GBR expression in Ca_v_3.2^−/−^ mice might therefore alter APP stability in these animals. Future studies will be necessary to unravel the potential functional interdependence between T-type VGCCs, the GABAergic system and APP and its relevance in the aetiopathogenesis of Alzheimer’s disease.

## Methods

### Study animals

In this study, Ca_v_3.2^+/−^ embryos (kindly provided by Kevin Campbell via MMRCC-Mutant Mouse Resource & Research Centers) were re-derived with C57BL/6J mice. All genotypes were obtained using random intra-strain mating. In total, eight Ca_v_3.2^+/+^ mice (all ♂, mean age: 124 ± 1 days) and eight Ca_v_3.2^−/−^ animals (all ♂, mean age: 129 ± 4 days) were analyzed electroencephalographically. Experimental animals were housed in clear Macrolon cages type II in groups of 3–4 with ad libitum access to drinking water and standard food pellets. Mice were maintained under controlled environmental conditions using the ventilated cabinet Model 9AV125P (Tecniplast, Germany) and the UniProtect cabinet (Bioscape, Germany) with the following settings: ambient temperature 21 ± 2 °C, relative humidity 50–60%, and conventional 12 h/12 h light/dark cycle starting at 5:00 a.m.

All animal experiments were carried out in accordance with the guidelines of the German council on animal care and experimental protocols were approved by the local institutional and national committee on animal care (State Agency for Nature, Environment and Consumer Protection; Landesamt für Natur, Umwelt und Verbraucherschutz, LANUV, Germany, AZ-Nr. 87-51.04.2010.A321). All animal experimentation was further conducted in line with the National Institute of Health Guide for the Care and Use of Laboratory Animals (NIH Publications No. 80-23) revised 1996 or the UK Animals (Scientific Procedures) Act 1986 and associated guidelines, or the European Communities Council Directive of 24th November 1986 (86/609/EEC) and September 22nd, 2010 (2010/63/EU). Specific effort was made to reduce the number of experimental animals and their suffering (3R strategy).

### Pre-surgical management of experimental animals and transmitter implantation

For pre-surgical preparation of experimental animals including selection of mouse lines, age and gender, anesthesia, temperature support, pain management, etc. please refer to our detailed descriptions^91,92^. Further details on the transmitter implantation are provided in Refs.^91–93^.

### Intrahippocampal electrode placement for electrohippocampal recordings

For intracerebral, deep EEG recordings from the hippocampal CA1 region, the differential electrode of the TA10ETA-F20 transmitter (Data Science International, DSI, USA), technical specifications: weight 3.9 g, volume 1.9 cc, input voltage range ± 2.5 mV, channel bandwidth (B) 1–200 Hz, nominal sampling rate (f) 1000 Hz (f = 5 B), temperature operating range 34–41 °C, warranted battery life 4 months, on–off mechanism magnetically actuated) was positioned at the following stereotaxic coordinates: (+)-lead, caudal − 2 mm, lateral of bregma 1.5 mm (right hemisphere), and dorsoventral (depth) 1.5 mm. The epidural reference electrode was positioned on the surface of the cerebellar cortex at the following stereotaxic coordinates: (−)-lead, bregma − 6 mm and lateral of bregma 1 mm (right hemisphere). For intracerebral recordings, the sensing lead of the transmitter was mechanically clipped to the deep electrode^91–93^. Notably, the deep tungsten electrodes (FHC, USA) are encapsuled with epoxylite with an impedance of 50–100 kΩ (measured at 1000 Hz) and a shank diameter of 250 μm. Epidural and intracerebral electrodes were fixed using glass ionomer cement (Kent Dental, Kent Express Ltd., UK) and the scalp was closed using over-and-over sutures (Ethilon, 6-0). Due to the body surface/body volume ratio, mice are highly susceptible to hypothermia. Thus, supplemental warmth was given to the animals during the entire period of anesthesia/surgical procedure and the first two days post implantation using a heating pad. A detailed description of the stereotaxic EEG electrode placement and transmitter implantation was previously given by Weiergräber and colleagues^91,92,94^. For peri- and post-operative pain management, carprofen (5 mg/kg, Rimadyl, Parke-Davis/Pfizer, Germany) was injected subcutaneously. Mice were given 10 days to fully recover after surgery. This recovery period was determined by the finding that no alterations in basic physiological/behavioral parameters such as water and food uptake, locomotion, surface and body core temperature, etc. could be detected between radiotransmitter-implanted, non-implanted, and sham-operated mice 10 days post surgery^48^.

### Confirmation of EEG electrode placement

To confirm that electrodes were positioned in the exact CA1 target area, brains were extirpated post mortem and fixed in 4% formaldehyde solution. Afterwards, brains were cut to 60 μm slices using a Vibroslice Tissue Cutter EMS 5000-MZ (Campden Instruments Limited, UK). Brain slices were stained with hematoxylin/eosin to visualize the branch canal (Supplementary Fig. 6). Mice in which EEG electrodes were not placed correctly in the defined target region were removed from the subsequent analysis.

### Radiotelemetric EEG data acquisition

In each experimental animal, the first 24 h baseline recording (R1) from the CA1 hippocampal region (electrohippocampogram) was obtained at day 10 post surgery.

This recovery period is based on the observation that 10 days post surgery no differences in physiological parameters between transmitter implanted, non-implanted, and sham-operated animals could be detected^48,95^.

A second 24 h long-term baseline recording (R2) was conducted at day 17 post implantation to check whether potential alterations in relative EEG frequency range power are robust over time or whether there are developmental changes^96–98^ (Fig. 1A).

In addition, two EEG recordings were performed following urethane injection (U1, U2) with 800 mg/kg i.p. (Sigma, Germany, freshly dissolved in 0.9% NaCl) at day 18 and 25 after implantation, respectively.

CA1 intrahippocampal EEG data were acquired using the Dataquest ART 4.2 software (Data Sciences International, DSI, USA). Note that EEG data were sampled at a nominal rate of 1000 Hz with no a priori filter cutoffs. Based on the Shannon-Nyquist theorem and limit, EEG frequency analysis was carried out up to 500 Hz (upper gamma range)^99^.

Besides biopotentials (such as EEG), the TA10ETA-F20 transmitter also provides temperature and activity data. As the transmitter was placed in a subcutaneous pouch on the back of the experimental animal in our setting, the recorded subcutaneous temperature values do not represent body core values. However, subcutaneous temperature data were shown to correlate with body core temperature under environmentally controlled conditions and can thus be compared within and between the individual genotypes^91–93,100,101^. Further note, that activity data are provided by the telemetry system in relative values (relative activity). These relative data represent activity in the horizontal plane and integrate trip distance, velocity and acceleration. Our EEG-activity correlation is based on a binary system with activity = 0 for the inactive state and activity > 0 for the active state. For details see also^91^.

### Analysis of electrohippocampographic EEG recordings

EEG data were exported to NeuroScore 3.2.9306-1 (Data Sciences International, DSI, USA) for further Fast-Fourier Transformation (FFT) based frequency analysis in the range of 0.5–500 Hz, including the following distinctive frequency bands: delta 1 (δ_1_, 0.5–4 Hz), delta 2 (δ_2_, 1–4 Hz), theta 1 (θ_1_, 4–8 Hz), theta 2 (θ_2_, 4–12 Hz), alpha (α, 8–12 Hz), sigma (σ, 12–16 Hz), beta 1 (β_1_, 12–30 Hz), beta 2 (β_2_, 16–24 Hz), beta 3 (β_3_, 16–30 Hz), gamma low (γ_low_, 30–50 Hz), gamma mid (γ_mid_, 50–70 Hz), gamma high (γ_high_, 70–100 Hz), gamma ripples (γ_ripples_, 80–200 Hz), and gamma fast ripples (γ_fast ripples_, 200–500 Hz)^91–93^. Note that we have included also a broader theta frequency band (theta-alpha band, θ_2_) in our analysis, based on the complex functional interdependence of hippocampal oscillatory activity^12,13,102–104^.

For FFT based analysis, the duration of the individual EEG epochs was determined as 2 s^91–93^. Mean relative EEG power (%) of the individual frequency ranges was calculated for the individual circadian stages, i.e., two dark cycles (DC1, DC2, 12 h each) and two light cycles (LC1, LC2, 12 h each), and 6 h post urethane 1 and 2 injection phases (U1, U2). Potential EEG artefacts were identified by both manual inspection of the EEG and the automated artefact detection tool of Neuroscore and were eliminated for EEG relative power analysis^91,92,94^.

Relative activity counts and temperature data were also analyzed for baseline (R1, R2) and post urethane recordings (U1, U2) mentioned above. Importantly, activity data (active state, i.e., activity units > 0, or inactive state, i.e., activity units = 0) during the conventional 12 h/12 h light/dark cycle (starting at 5:00 a.m.) were correlated with the relative EEG power of the individual frequency bands from the hippocampal CA1 deflection.

Data were statistically analyzed and displayed as mean ± SEM. Statistics for FFT based frequency analysis were performed using multiple Student’s t-test, corrected for multiple comparison using the Holm-Sidak approach (*p < 0.05; **p < 0.01; ***p < 0.001). Statistics and graphical representations were conducted using GraphPad Prism 6 for Windows (Graphpad Software, Inc., USA).

### Quantitative real time PCR (qPCR)

Transcriptome analysis of hippocampi from Ca_v_3.2^+/+^ and Ca_v_3.2^−/−^ mice suggested various gene candidates that could be functionally related to altered theta genesis^49^. To investigate the molecular mechanisms involved in the generation of hippocampal EEG oscillations in Ca_v_3.2 deficient mice, hippocampal transcript levels of potential gene candidates, i.e. dynein light chain Tctex-Type 1 (Dynlt1b), Neuronatin (Nnat), LLP homolog, long-term synaptic facilitation (Aplysia) (Llph), ATP synthase, H^+^ transporting, mitochondrial F_0_ complex, subunit G (Atp5) and 5-hydroxytryptamine receptor 2C (Htr2c) were analyzed using quantitative Real-Time PCR (RT-PCR, qPCR).

In a second approach, qPCR analysis of selected gene candidates of the GABAergic system was performed including GABA A receptor delta subunit (Gabrd), GABA A receptor gamma 2 subunit (Gabrg2), GABA B_1_ receptor subunit (Gabbr1) and GABA B_2_ receptor subunit (Gabbr2). These subunits were selected for the following reasons: In mammals, sequences of six α, three β, three γ, one δ, three ρ, one ε, one π and one θ GABA A receptor subunits have been described^105–108^. A majority of GABA A receptor subtypes contains α, β and γ subunits with a stoichiometry of 2α.2β.1γ^105,109^. Notably, most GABA A receptors containing the γ2 subunit tend to form clusters at the postsynaptic membrane, whereas GABA A receptors incorporating the δ subunit seem to be exclusively localized extrasynaptically^110–113^. We have thus checked for the GABA A δ and γ subunits in our qPCR study. Concerning GABA B receptors, we analyzed both GABA B1 and GABA B2 subunits as they regulate both pre- and postsynaptic activity^114–118^.

Finally, Ca_v_3.1 (Cacna1g) and Ca_v_3.3 (Cacna1i) were analyzed to check for potential alterations in other LVA T-type Ca^2+^ channel transcript levels. Forward and reverse primer sequences of gene candidates are displayed in Table 1.

Total RNA was extracted from the hippocampus of male Ca_v_3.2^+/+^ animals (mean age: 19.32 ± 0.44 weeks, n = 8) and male Ca_v_3.2^−/−^ mice (mean age: 20.43 ± 0.41 weeks, n = 8). Additionally, the hippocampus of a female Ca_v_3.2^+/+^ mouse aged 24.14 weeks was dissected, which served as a calibrator in our study. The calibrator contains RNA from the genes of interest and the housekeeping genes and is used on every PCR plate for every gene tested.

First, hippocampal tissue was dissected in RNAprotect Tissue Reagent (Qiagen, Germany) and snap-frozen in liquid nitrogen. Total hippocampal RNA was extracted using RNeasy Lipid Tissue Mini Kit (Qiagen, Germany) including DNA degradation (additional DNase digestion step). Quality and quantity of the extracted RNA was evaluated using Nanodrop (Nanodrop 1000, Thermo Fisher Scientific, Germany). To obtain a 50 µl cDNA volume, 1 µg of total RNA from each animal was reversely transcribed in a two-step RT-PCR approach using both anchored-oligo (dT)_18_ and hexamer primers (Transcriptor First Strand cDNA Synthesis Kit, Roche, Switzerland). Gene candidates were tested in triplicates in each animal using 2 µl cDNA as a template. In addition, a triplicate of calibrator cDNA was carried out for normalization of potential inter-run variations. Duplicates of two negative controls, i.e., no template controls and no reverse transcriptase controls were performed to exclude false positive results. Note, that mice used for qPCR analysis did not undergo transmitter implantation and EEG recordings.

qPCR was conducted in a Light Cycler 480 System (Roche, Switzerland) using the following protocol: 95 °C (10 min, pre-incubation step); 95 °C (10 s, denaturation step); 60 °C (20 s, annealing step); 72 °C (30 s, extension step), 35 cycles. This protocol was applied to all tested primer pairs (Table 1). SYBR Green 1 Master (Roche, Switzerland) was used for signal detection and the specificity of amplification was evaluated by melting curve analysis.

The CP values received from the Light Cycler 480 Software (Roche, Switzerland) were exported to qBase + software (Biogazelle, Belgium) and analyzed based on a delta-Cq quantification model with qPCR efficiency correction, reference gene normalization considering the reference target stability of the selected housekeeping genes (HPRT, β-actin) and inter-run calibration^119^. The results were characterized as Calibrated Normalized Relative Quantity (CNRQ) and statistically analyzed using the Mann–Whitney test.

## Supplementary Information

Supplementary Information.

## Data Availability

Relative EEG power data of this study are available at Mendeley data (doi: 10.17632/x53km5sby6.1; 10.17632/x53km5sby6.1).

## Abbreviations

AS: Active state
CA: Cornu ammonis
CNRQ: Calibrated normalized relative quantity
DAG: Diacylglycerole
DC: Dark cycle
DHP: Dihydropyridine
EC: Entorhinal cortex
GABA: Gamma-aminobutyric acid
GPCR: G-protein coupled receptor
GT: Genotype
HVA: High voltage-activated
i.p.: Intraperitoneal
LC: Light cycle
LTP: Long-term potentiation
LVA: Low voltage-activated
mAChR: Muscarinic acetylcholine receptor
MS/DBB: Medial septum/diagonal band of Broca
MS: Medial septum
MVA: Mid voltage-activated
NAS: Non-active state
PKC: Protein kinase C
PSD: Power spectrum density
qPCR: Quantitative polymerase chain reaction
REM: Rapid eye movement
R-type: “Resistant” type Ca^2^^+^ channel
SEM: Standard error of the mean
SK: Small-conductance potassium channel
TM: Transmitter
T-type: “Transient” type Ca^2^^+^ channel
VGCC: Voltage-gated Ca^2^^+^ channel
