# Supplementary material for: Enhanced hippocampal type II theta activity AND altered theta architecture in mice lacking the Cav3.2 T-type voltage-gated calcium channel
Source: Sci Rep. 2021 Jan 13;11:1099. doi: 10.1038/s41598-020-79763-4 (PMC7806756; doi:10.1038/s41598-020-79763-4)

Supplementary Information

Enhanced Hippocampal Type II Theta Activity AND Altered Theta Architecture in Mice Lacking the $Ca_V3.2$ T-Type Voltage-Gated Calcium Channel

Muhammad Imran Arshaad[a], Magdalena Elisabeth Siwek[a], Christina Henseler[a], Johanna Daubner[a], Dan Ehninger[b], Jürgen Hescheler[c], Agapios Sachinidis[c], Karl Broich[d], Anna Papazoglou[a] and Marco Weiergräber [a,*]

[a] Experimental Neuropsychopharmacology, Federal Institute for Drugs and Medical Devices (Bundesinstitut für Arzneimittel und Medizinprodukte, BfArM),
Kurt-Georg-Kiesinger-Allee 3, 53175 Bonn, Germany

[b] Molecular and Cellular Cognition, German Center for Neurodegenerative Diseases (Deutsches Zentrum für Neurodegenerative Erkrankungen, DZNE), Sigmund-Freud-Str. 27, 53127 Bonn, Germany

[c] Institute of Neurophysiology, University of Cologne, Faculty of Medicine, Robert-Koch-Str. 39, 50931 Cologne, Germany

[d] Federal Institute for Drugs and Medical Devices (Bundesinstitut für Arzneimittel und Medizinprodukte, BfArM), Kurt-Georg-Kiesinger-Allee 3, 53175 Bonn, Germany

* To whom correspondence should be addressed:
Prof. Dr. nat. med. Dr. med. Marco Weiergräber (MD, PhD)
Experimental Neuropsychopharmacology, Federal Institute for Drugs and Medical Devices (Bundesinstitut für Arzneimittel und Medizinprodukte, BfArM)
Kurt-Georg-Kiesinger-Allee 3, 53175 Bonn, Germany
Phone: +49 228 99307 4358
Fax: +49 228 99307 3896
Email: Marco.Weiergraeber@bfarm.de

Supplementary figure 1: EEG power analysis during the active light cycle state (24 h long-term recording R2) in $Ca_v3.2^{+/+}$ and $Ca_v3.2^{-/-}$ mice. Relative EEG power (%) for $Ca_v3.2^{+/+}$ and $Ca_v3.2^{-/-}$ mice is displayed for the individual frequency ranges (**A-E)**. A significant increase for α and σ relative power was observed in $Ca_v3.2^{-/-}$ mice (**C**).

Supplementary figure 1 (Arshaad et al., 2020)

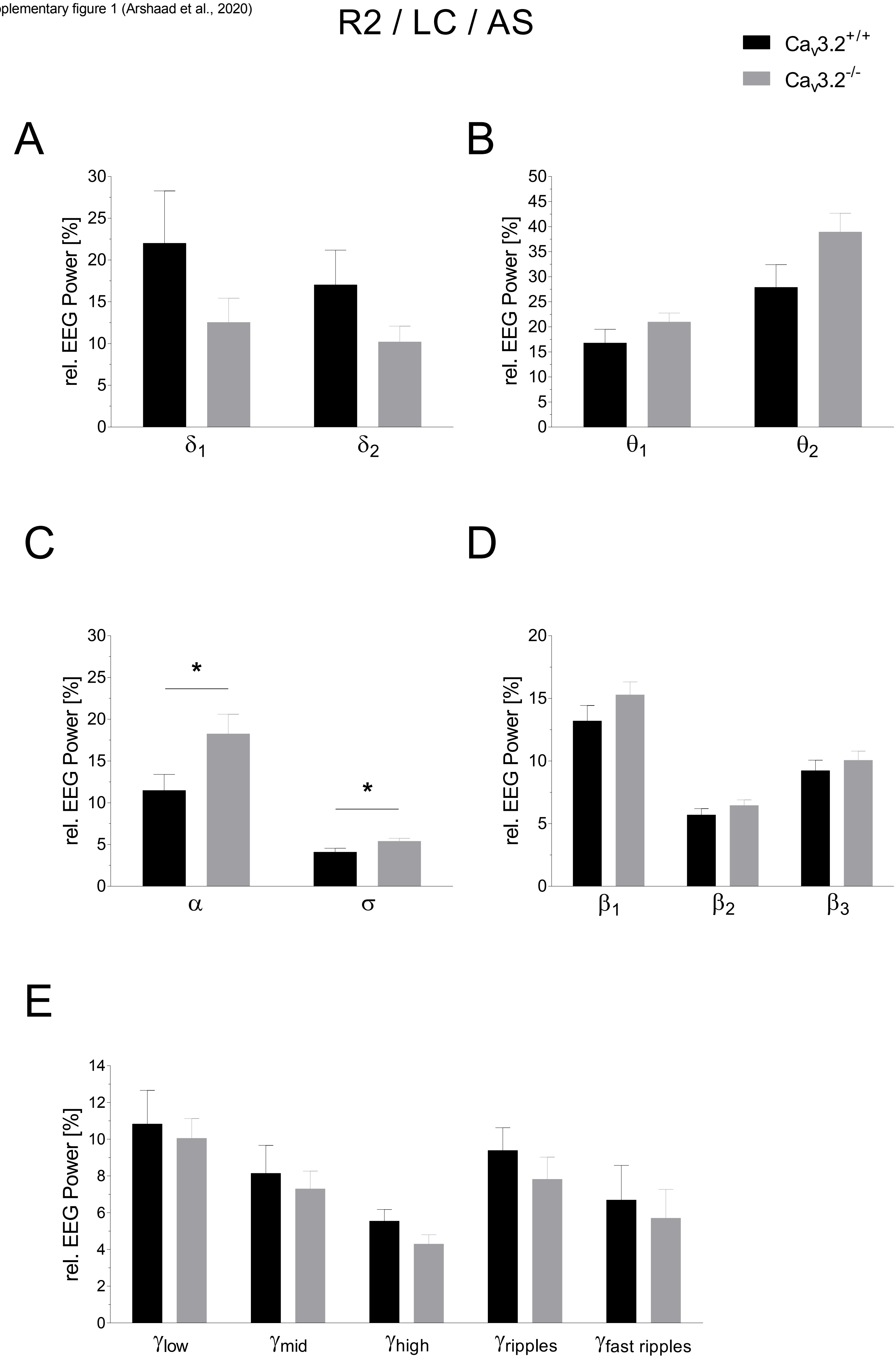

Supplementary figure 2: EEG power analysis during the non-active light cycle state (24 h long-term recording R2) in $Ca_v3.2^{+/+}$ and $Ca_v3.2^{-/-}$ mice. Relative EEG power (%) for the $Ca_v3.2^{+/+}$ and $Ca_v3.2^{-/-}$ is displayed for the individual frequency ranges (**A-E**). In $Ca_v3.2^{-/-}$ mice, a significant increase in θ_2 and α was observed compared to controls (**B, C**). As for R1, these results point to a functional role of $Ca_v3.2$ in type II theta genesis.

Supplementary figure 2 (Arshaad et al., 2020)

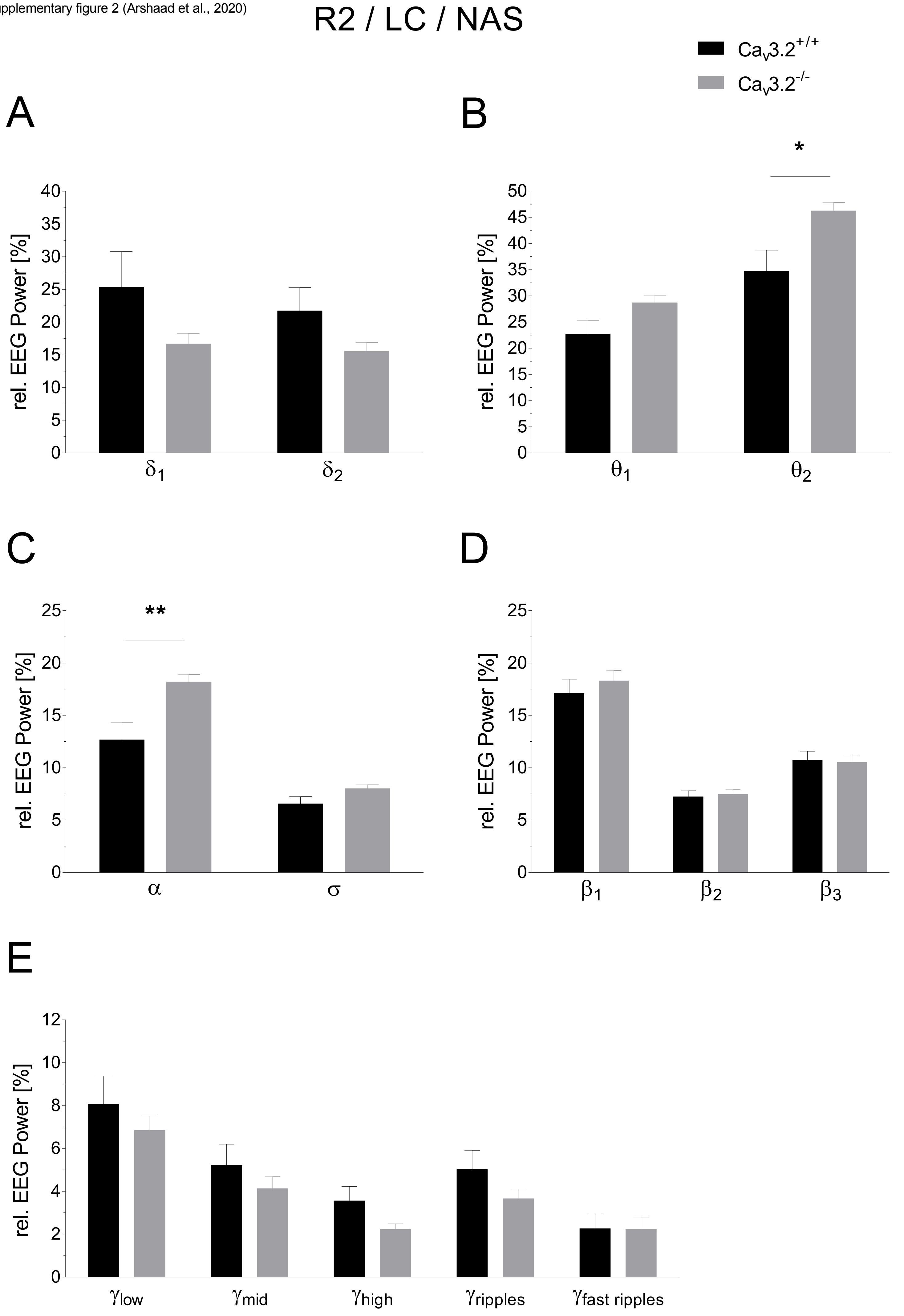

Supplementary figure 3: EEG power analysis during the active dark cycle state (24 h long-term recording R2) in $Ca_v3.2^{+/+}$ and $Ca_v3.2^{-/-}$ mice. Relative EEG power (%) for $Ca_v3.2^{+/+}$ and $Ca_v3.2^{-/-}$ mice is displayed for the individual frequency ranges (**A-E**). No significant alterations were detected.

Supplementary figure 3 (Arshaad et al., 2020)

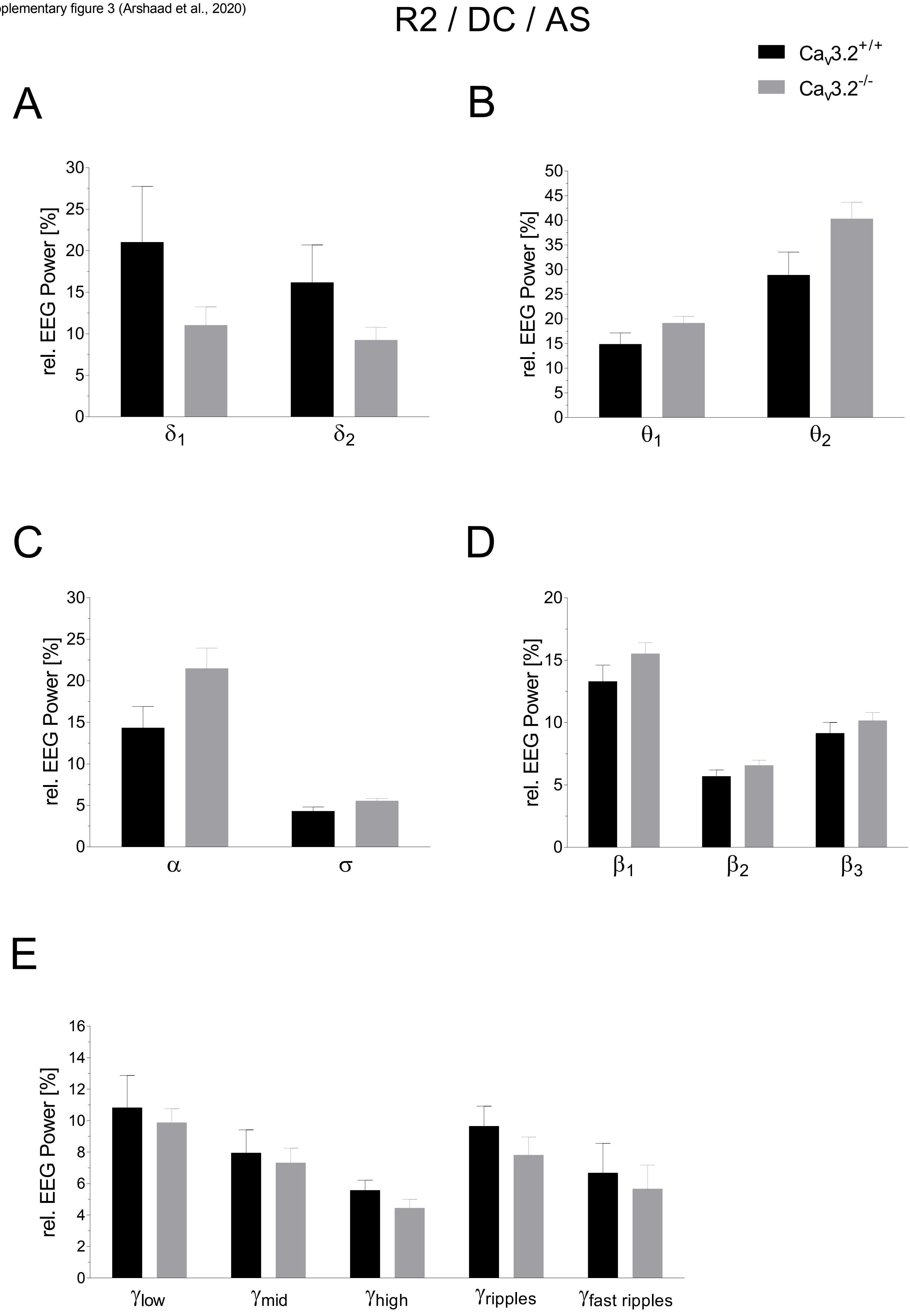

Supplementary figure 4: EEG power analysis during the non-active dark cycle state (24 h long-term recording R2) in $Ca_v3.2^{+/+}$ and $Ca_v3.2^{-/-}$ mice. Relative EEG power (%) for $Ca_v3.2^{+/+}$ and $Ca_v3.2^{-/-}$ mice is displayed for the individual frequency ranges (**A-E**). A significant increase in relative θ_2 and α power was observed in $Ca_v3.2^{-/-}$ mice (**B, C**). As for R1, these results point to a functional role of $Ca_v3.2$ in type II theta genesis.

Supplementary figure 4 (Arshaad et al., 2020)

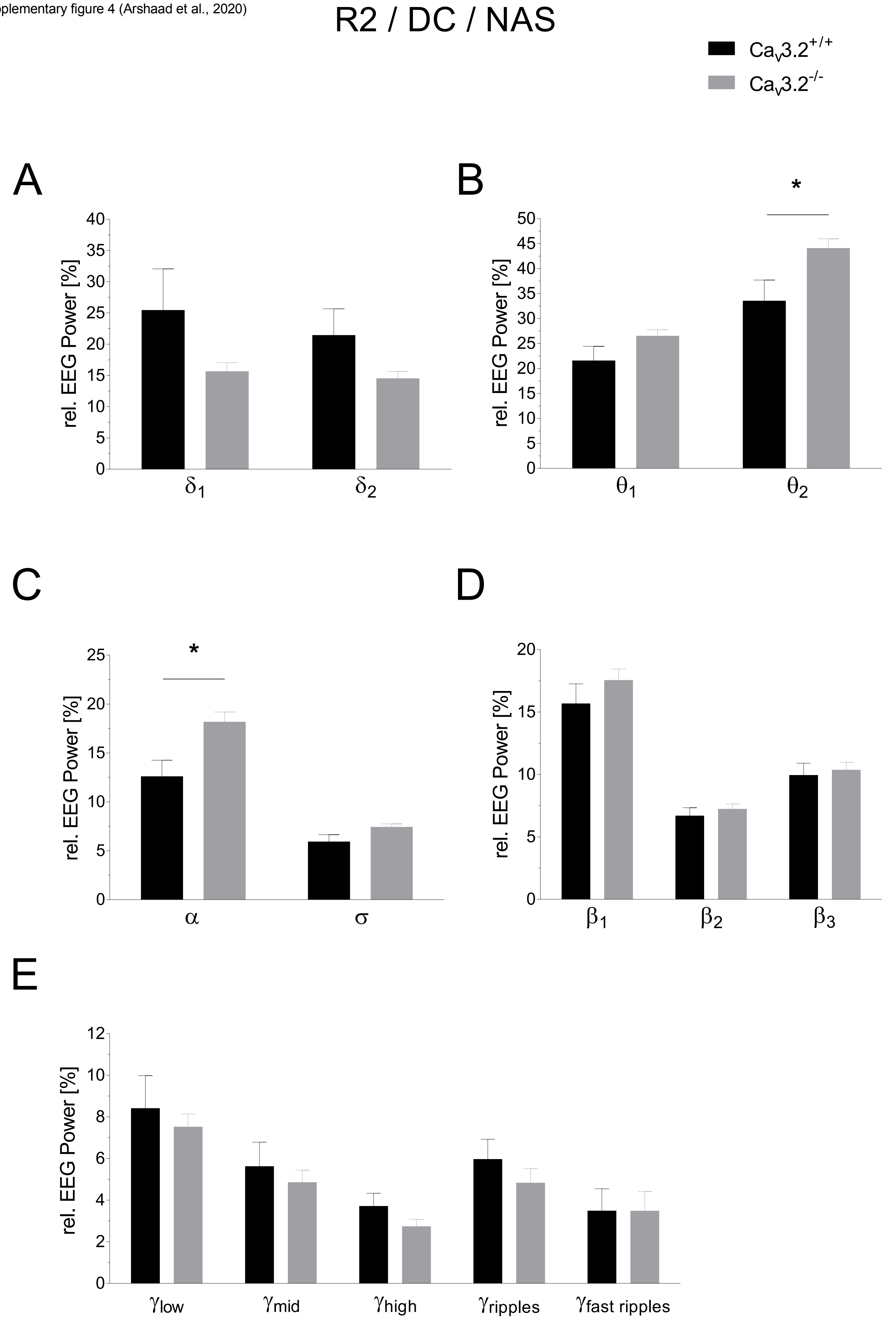

Supplementary figure 5: Frequency analysis in $Ca_v3.2^{+/+}$ and $Ca_v3.2^{-/-}$ mice following urethane injection (U2). Relative EEG power (%) for the $Ca_v3.2^{+/+}$ and $Ca_v3.2^{-/-}$ is displayed for the individual frequency ranges (**A-E**). Note that urethane administration results in a significant increase of θ_1, θ_2 and α relative power compared to controls (**B, C**). These findings support the notion that $Ca_v3.2$ functionally contributes to type II theta genesis.

Urethane 2

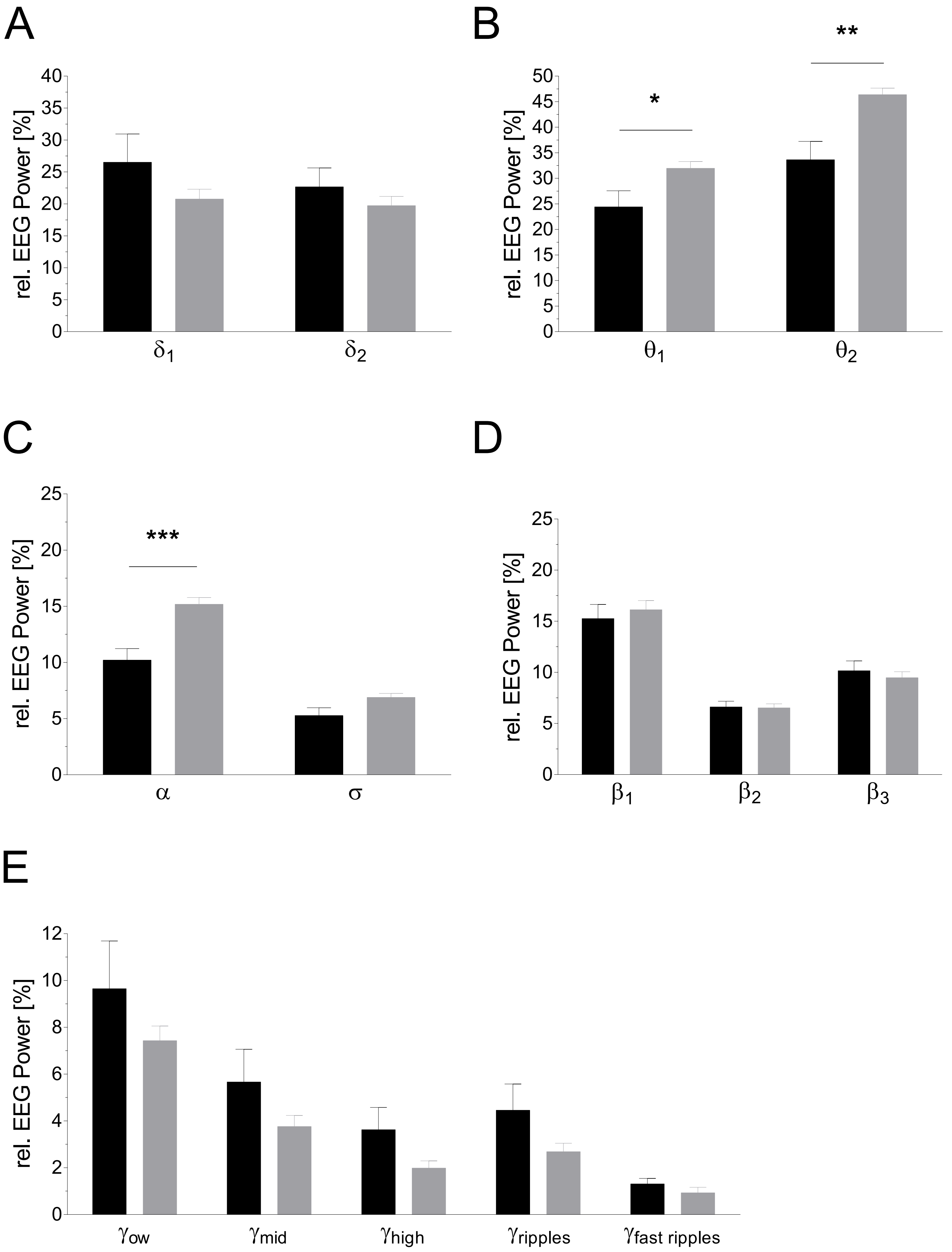
A
40
35
30
25
20
15
10
5
0
rel. EEG Power [%]
δ1
δ2
B
**
*
50
45
40
35
30
25
20
15
10
5
0
rel. EEG Power [%]
θ1
θ2
C
25
20
15
10
5
0
***
rel. EEG Power [%]
α
σ
D
25
20
15
10
5
0
rel. EEG Power [%]
β1
β2
β3
E
12
10
8
6
4
2
0
rel. EEG Power [%]
γow
γmid
γhigh
γripples
γfast ripples

Supplementary figure 6: Verification of deep electrode placement. Following deep electrode removal, the extirpated brain is cut into coronal sections. The branch canal visualizes the prior electrode placement targeting the pyramidal layer of the CA1 region. Note that the branch canal is enlarged due to formaldehyde mediated shrinking processes (cc, corpus callosum; Cx, cortex; DG, dentate gyrus; gl, granule cell layer; ml, molecular layer; pl, pyramidal cell layer; pml, polymorphic layer; slm, stratum lacunosum moleculare; so, stratum oriens; sr, stratum radiatum).

Supplementary figure 6 (Arshaad et al., 2020)

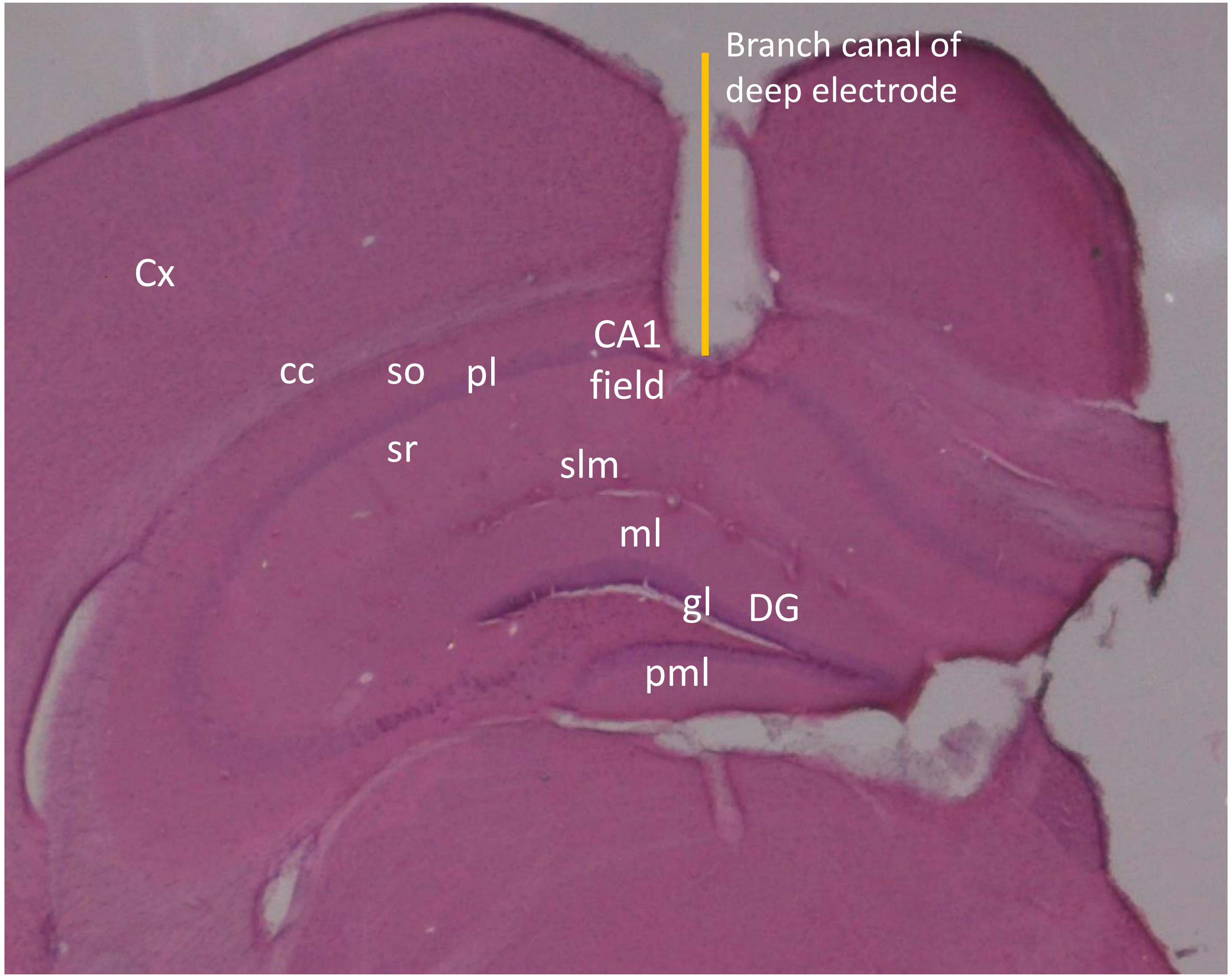

Supplement: Supplementary file 1 — Supplementary Information. [file 41598_2020_79763_MOESM1_ESM.pdf]
